# A Multi-Object Tracking Method for Dairy Cows in Intensive Farming Scenarios

**DOI:** 10.3390/ani16182884

**Published:** 2026-09-13

**Authors:** Zhihua Diao, Zhichao Huang, Jiangbo Li, Jinpeng Cheng, Suna Zhao, Baohua Zhang

**Affiliations:** 1 College of Electrical Information Engineering, Zhengzhou University of Light Industry, Zhengzhou 450002, China; diaozhua@163.com (Z.D.); 19838067829@163.com (Z.H.); zsnzsn1221@163.com (S.Z.); 2 Intelligent Equipment Research Center, Beijing Academy of Agriculture and Forestry Sciences, Beijing 100000, China; lijb@nercita.org.cn; 3 College of Agronomy, Henan Agricultural University, Zhengzhou 450046, China; jpeng_cheng@163.com; 4 College of Artificial Intelligence, Nanjing Agricultural University, Nanjing 211800, China

**Keywords:** dairy cows, precision livestock farming, multi-object tracking, YOLOv10s

## Abstract

To better analyze the health and welfare of individual dairy cows, this study proposes BR-Tracker, a multi-object tracking method designed for dense monitoring environments to address missed detections, tracking failures, and frequent identity switches. In the object detection stage, a Receptive Field Attention Downsampling (RFADown) module is introduced into the neck network of YOLOv10s to effectively process the locally visible regions of occluded cows by dynamically adjusting the receptive field. An improved Partial Bi-Level Routing Attention (PBRA) module is incorporated into the backbone network to simultaneously extract global and local features, while a Spatial Pyramid Pooling with Efficient Layer Aggregation Network (SPPELAN) module is adopted to enhance multi-scale feature aggregation. In the object tracking stage, an MPDIoU-based matching algorithm is designed to improve matching accuracy and the reliability of trajectory association. The dataset contains 15,420 images for object detection and 130 independent videos for multi-object tracking, of which 40 videos are selected for the final tracking evaluation. Experimental results show that BR-YOLOv10s achieves a precision, recall, and mean average precision (mAP) of 95.7%, 90.3%, and 95.2%, respectively. Compared with the YOLOv10s-ByteTrack baseline, the proposed method improves HOTA, MOTA, MOTP, and IDF1 by 4.4, 5.7, 3.0, and 6.1 percentage points, respectively, while reducing identity switches by 23.19%. In addition, the tracker achieves a processing speed of 43.2 FPS. These results demonstrate that the proposed method can effectively perform real-time detection and tracking of densely distributed dairy cows in complex intensive farming environments.

## Simple Summary

Modern dairy farming is gradually adopting intelligent video surveillance systems to support the monitoring of dairy cow health and welfare. However, in intensive indoor barns, frequent mutual occlusion among cows severely limits the reliability of traditional vision algorithms for continuously tracking individual identities. To address this problem, this study proposes an improved real-time visual tracking method specifically for densely distributed dairy cow herds. First, the core visual detection model is optimized to enhance the system’s ability to capture locally visible features of occluded cows. Second, the target matching mechanism is improved, enabling the system to accurately associate current targets with historical trajectories even when the visual contours of cows change due to crowding and occlusion. Tests in real ranch environments show that the proposed method achieves better tracking stability than existing mainstream systems. Notably, compared with the YOLOv10s-ByteTrack baseline, the proposed method reduces ID switches by 23.19%, achieves a processing speed of 43.2 FPS, and maintains favorable performance under both daytime and nighttime conditions. This study provides a reliable non-contact monitoring solution for the analysis of daily dairy cow behaviors and offers important technical support for subsequent behavioral analysis, disease early warning and precision dairy farming management.

## 1. Introduction

With the continuous development of the economy, the demand for high-protein dairy products has gradually increased, and the dairy farming industry has consequently received growing attention. Consequently, the smart breeding industry for dairy cows has gained significant importance. Among its various components, intelligent monitoring equipment is crucial in assessing cow health, enhancing cow welfare, reducing labor costs, and increasing breeding efficiency [1,2,3]. Numerous studies have indicated that monitoring the daily behavior and activities of dairy cows can greatly assist in disease analysis and diagnosis, thereby improving production efficiency, welfare, and health status of dairy cows [4,5,6]. Existing intelligent monitoring devices are classified into contact-based and non-contact-based types [7]. Contact-based devices include many kinds of hardware, such as GPS collars connected to the network developed based on the Internet of Things (IoT) [8] and signal transmitters installed on the ears of dairy cows [9]. However, contact-based devices incur high usage costs and may interfere with the daily activity decisions of dairy cows [10]. In addition, existing wearable devices still face practical limitations, including insufficient integration of multiple sensors, limited interoperability with existing pasture management systems, inadequate energy autonomy and high operating costs. These factors may hinder their wide-scale application at the pasture level, especially in small and medium-sized pastures [11]. In contrast, although the detection accuracy of non-contact monitoring equipment is lower than that of contact-type equipment, it has advantages, including low cost, easy deployment and no disturbance to the normal activities of individual cows [12,13,14].

Although existing studies have achieved good progress in livestock and poultry object detection and multi-object tracking, their application to semi-enclosed intensive dairy farming environments still faces some problems that require further resolution. (1) In terms of object detection, a large number of cows usually coexist in practical cowsheds, the distances between different cows and monitoring equipment vary, and mutual occlusion between targets inevitably occurs [15]. This not only causes scale differences in cow targets in images but also leaves only limited locally visible regions for partially occluded targets. During feature extraction and spatial downsampling, such locally effective information is easily weakened, thereby increasing the risk of missed detection of occluded targets and confusion between adjacent targets. Existing studies have shown that multi-scale context modeling and adaptive feature aggregation can enhance feature representation capability and detection robustness in complex scenarios [16]. Existing feature selection mechanisms also improve feature expression from different perspectives. For example, Selective Kernel Attention (SK) achieves scale selection by adaptively weighting convolutional branches of different scales [17], Convolutional Block Attention Module (CBAM) weights channel and spatial features [18], and Bi-Level Routing Attention (BRA) performs selective information aggregation based on regional correlation [19]. However, in dense dairy cow detection scenarios, it is necessary not only to adapt to target scale changes but also to balance the retention of locally visible information of occluded targets and the effective aggregation of relevant context information. Therefore, how to improve multi-scale target representation capability, reduce local feature loss of occluded targets, and enhance feature discrimination capability between dense targets remains a problem that requires further resolution. (2) In terms of object tracking, existing studies have made improvements from the aspects of appearance feature representation, individual identity recognition, occlusion handling and trajectory matching respectively. Han et al. [20] and Li et al. [21] used wide residual networks to extract dairy cow appearance features and combined improved DeepSort to achieve individual tracking; Pretto et al. [22] used the Optical Character Recognition (OCR) algorithm to recognize dairy cow ear tag numbers to determine individual identities; Guo et al. [23] enhanced dairy cow spatial information and appearance feature expression by introducing coordinate attention (CA) and Vision Transformer (ViT). To address occlusion and trajectory association problems, Zhou et al. [24] combined optical flow with ByteTrack to improve tracking performance under occlusion, Lu et al. [25] introduced a center distance matching mechanism into ByteTrack to reduce identity switches, Mg et al. [26] adopted the Customized Tracking Algorithm (CTA) to search for lost IDs during re-identification, and Zheng et al. [27] expanded the matching space between detection results and trajectories through the Cascaded-Buffered IoU (C-BIoU). Although the above methods have improved tracking performance from different perspectives, most relevant studies have been conducted in open-air farming environments or scenarios with relatively sparse cow distribution. In semi-enclosed intensive cowsheds, cows are more densely distributed, and individual appearances are similar. Frequent occlusion and target overlap not only reduce the discrimination capability of appearance features but also cause changes in the position, size and shape of detection boxes. For methods that mainly perform trajectory association based on the overlap degree between detection boxes and prediction boxes, when the overlap relationship of bounding boxes changes significantly, the matching reliability tends to decrease, thereby increasing the risks of erroneous associations, trajectory interruption and identity switches. Therefore, how to improve the matching reliability between detection results and predicted trajectories under dense occlusion conditions is also a problem that requires further resolution at present.

To address the above problems, this study proposes a multi-object tracking method for densely distributed dairy cows in semi-enclosed intensive farming environments and makes improvements from two perspectives, namely object detection and object tracking. (1) In terms of object detection, to address the problem of target scale changes caused by different shooting distances, this study introduces the Spatial Pyramid Pooling with Efficient Layer Aggregation Network (SPPELAN) module into the YOLOv10s [28] backbone network to enhance the extraction and fusion capability of multi-scale features by aggregating context information within different receptive field ranges. To address the problem wherein locally visible features under occlusion conditions are easily weakened or lost during spatial downsampling, the RFADown module is proposed to improve the downsampling structure in the neck network so as to enhance the retention capability of locally effective features. To address the problem of insufficient target feature discrimination capabilities under dense distribution and partial occlusion conditions, the PBRA module integrating PSA and Bi-Level Routing Attention (BRA) is introduced to enhance the utilization of relevant context features through regional selective information aggregation and improve the feature representation and discrimination capability of dense and partially occluded cows. Through the above improvements, the BR-YOLOv10s detection model is constructed to improve detection performance for cow targets in dense occlusion scenarios. (2) In terms of object tracking, to address the problem wherein occlusion and target interleaving cause changes in the position, size and shape of detection boxes, thereby reducing the reliability of IoU-based trajectory matching, this study adopts MPDIoU to optimize the trajectory association mechanism and designs a dairy cow multi-object tracker, BR-Tracker, accordingly. While considering the overlap relationship between detection boxes and prediction boxes, MPDIoU further introduces spatial distance information between corresponding corner points, thereby improving the matching reliability when the position and shape of target boxes change [29], and reducing erroneous associations and identity switches under partial occlusion conditions. To verify the effectiveness of the proposed method in practical intensive farming scenarios, this study collects a dataset under real dairy farm conditions and conducts multiple groups of object detection and multi-object tracking experiments.

## 2. Materials and Methods

### 2.1. Dataset Construction

**Figure 1:**
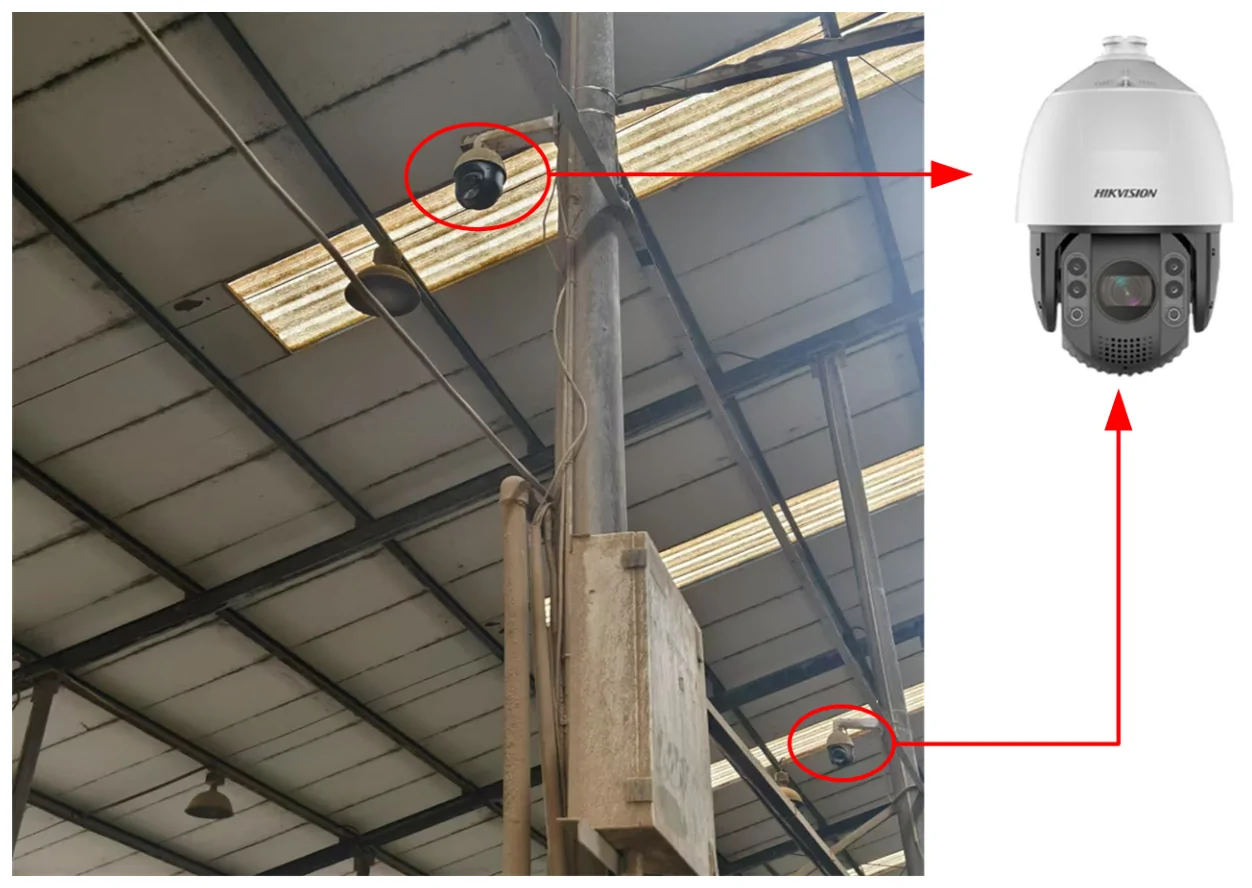
Camera position diagram.

**Figure 2:**
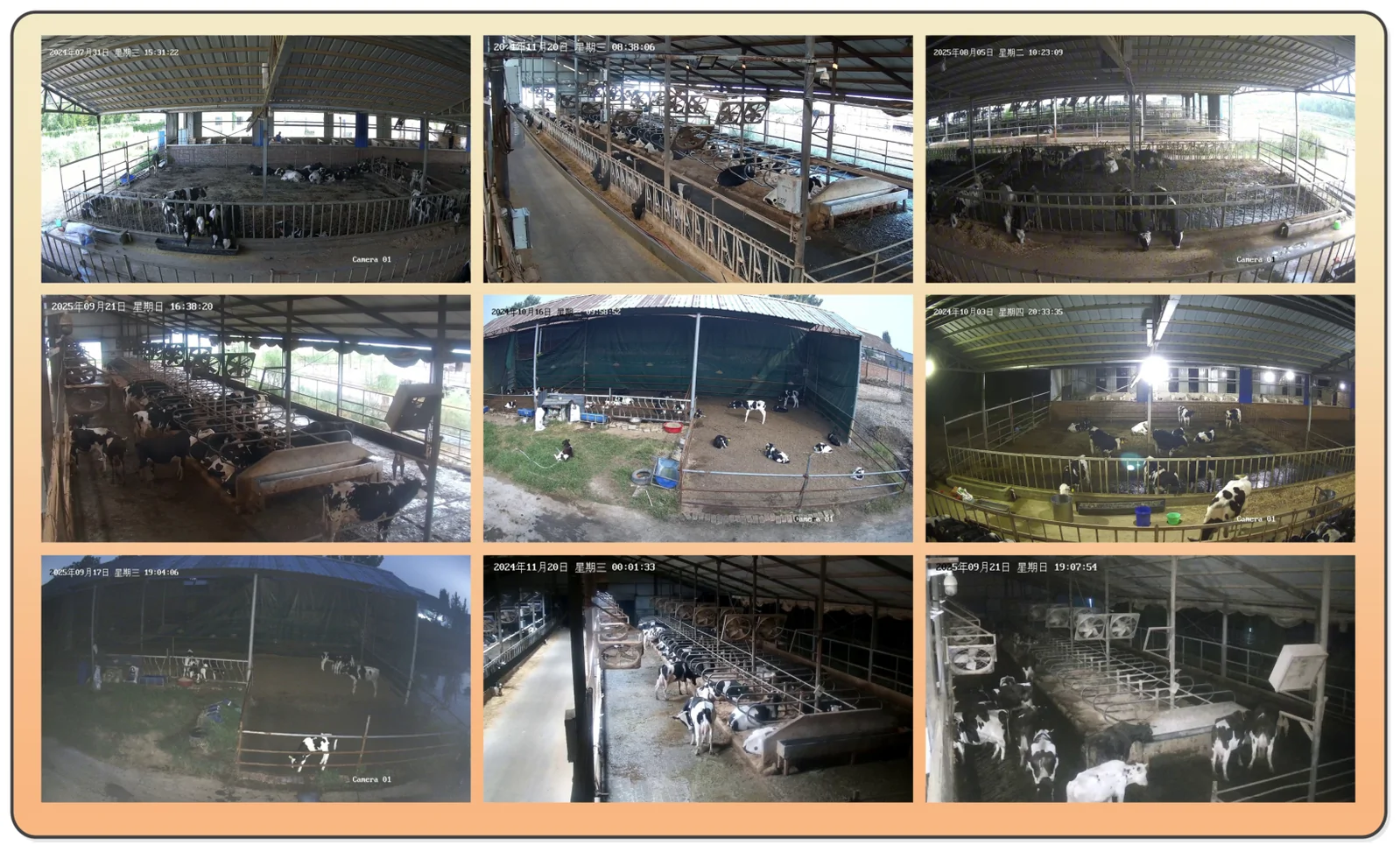
Monitoring scenarios of cattle under different day and night conditions. (In the original surveillance timestamps, “年”, “月”, “日”, and “星期” denote the year, month, day, and day of the week, respectively).

The data used in this study were collected from two dairy farms in Henan Province, China: the dairy breeding base in Yuanyang County, Xinxiang City, and a dairy farm in Qi County, Kaifeng City. A total of 13 Hikvision (Hangzhou, China) fixed surveillance cameras were installed in different cowsheds at an approximate height of 4.5 m. The cameras continuously recorded the dairy cows throughout the day from a downward viewing angle. Each cowshed housed a different group of Holstein dairy cows. All surveillance videos were recorded at a resolution of 1920 × 1080 pixels and a frame rate of 30 FPS. The camera layout and representative monitoring scenarios under daytime and nighttime conditions are shown in Figure 1 and Figure 2, respectively.

The dataset consisted of two components: an object detection dataset and a multi-object tracking (MOT) dataset. For the object detection dataset, the original surveillance videos were grouped by cowshed and used as the basic units for dataset partitioning. The training, validation, and test sets were divided at a ratio of 8:1:1, ensuring that the same cowshed and individual cows did not appear in different subsets. Considering the high temporal correlation between adjacent frames, images were extracted from each approximately 2 h original video at intervals of 10 s. This strategy reduced data redundancy and annotation workload while retaining variations in cow posture, occlusion status, and spatial distribution. Highly similar frames were subsequently removed manually, and samples showing meaningful differences were retained. After screening, approximately 600 frames per video were retained for annotation. A total of 15,420 images were obtained, and cow bounding boxes were annotated using LabelImg (version 1.8.6). The MOT dataset consisted of 130 original surveillance videos independent of the object detection dataset. Each video was approximately 5 min long. The dataset covers daytime and nighttime scenarios with a ratio of approximately 6:4, from which 40 videos are randomly selected for standard MOT evaluation. When semi-automatic annotation is performed using DarkLabel (version 2.4), a pretrained baseline detection model is first used to generate initial bounding boxes and preliminary cross-frame trajectory associations for all frames. Subsequently, annotators manually correct missed detections and false detections, calibrate bounding box positions, and fix ID association errors in the DarkLabel interface. Each annotated video contains approximately 9000 annotated frames. The annotation process was jointly completed by six domain experts with experience in dairy cow object detection and multi-object tracking. Four experts performed the initial annotations, one expert conducted cross-checking, and one senior expert was responsible for final review and adjudication of ambiguous samples. For cows that were occluded or reappeared after temporarily leaving the field of view, their identities were determined by jointly considering spatial position, motion trajectory, and appearance information in consecutive frames before and after the interruption. The original identity number was retained when a reliable correspondence could be established. Standardized annotation guidelines, cross-checking, and final expert review were used to ensure the consistency and accuracy of the annotations.

All data in this study were collected through non-contact video monitoring under routine farm management conditions. The surveillance process did not involve any invasive procedures, physical restraint, or direct interference with the dairy cows. Therefore, no additional stress or harm was caused to the animals during data acquisition. Permission for video data collection was obtained from the participating dairy farms, and all procedures complied with relevant animal welfare and ethical guidelines for agricultural research.

### 2.2. Detection Method

#### 2.2.1. Detector

**Figure 3:**
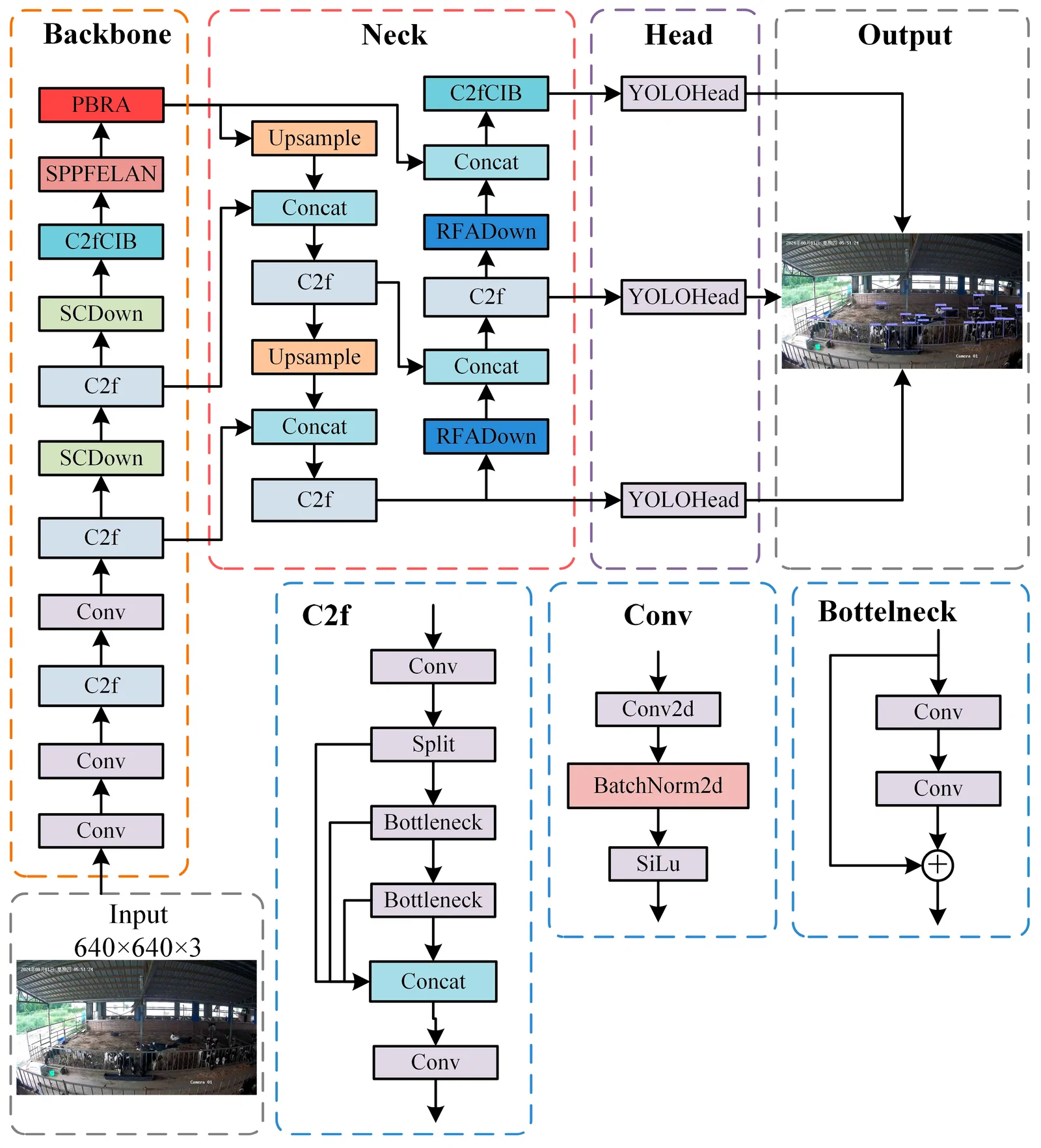
Structure diagram of the improved YOLOv10s network. Arrows indicate feature flow; ⊕ denotes element-wise addition. (The Chinese timestamp labels “年”, “月”, “日”, and “星期” denote the year, month, day, and day of the week, respectively).

This study adopts YOLOv10s, proposed by the research team of Tsinghua University, as the baseline detection network. YOLOv10s achieves end-to-end detection without Non-Maximum Suppression (NMS) during the inference phase through a consistent dual assignment strategy. Meanwhile, the C2fCIB module is applied in deep feature layers with rich semantic information, and a lightweight classification head is configured to balance feature extraction capability and computational efficiency. On this basis, aiming at problems that include severe cow occlusion, prominent target scale differences, and easy loss of locally visible features during downsampling in intensive breeding scenarios, this paper makes targeted improvements based on the original structure of YOLOv10s. As shown in Figure 3, the improved network consists of three components: backbone, neck and head. The main improvements are made to the deep feature aggregation module and attention module in the backbone, as well as the downsampling structure in the neck.

In the backbone, the original Spatial Pyramid Pooling-Fast (SPPF) module is replaced with the SPPELAN module. To avoid ambiguity in layer numbering, the layer numbers described in this paper are consistent with those in the model configuration file and adopt a zero-based indexing method. The SPPELAN module is arranged at the deep feature aggregation layer numbered 9 in the backbone, with a channel configuration of 1024 and a max pooling kernel size of 5 × 5. When the input image size is 640 × 640 pixels, both the input and output feature map sizes of this module are 512 × 20 × 20. It enhances the multi-scale information fusion capability of deep features without altering the number of feature map channels and spatial resolution. Subsequently, PBRA is adopted to replace the original Partial Self-Attention (PSA) module in the attention layer numbered 10. It has a channel configuration of 1024 and the same input and output feature map size of 512 × 20 × 20, so as to enhance the network’s feature representation capability for densely distributed and partially occluded cow targets. The neck adopts the Path Aggregation Network (PAN) structure to realize bidirectional fusion of shallow spatial detail features and deep semantic features. To reduce the loss of local feature information during downsampling, RFADown modules with channel configurations of 256 and 512 are used to replace the original Spatial-Channel Decoupled Downsampling (SCDown) module at the scale transformation layers numbered 17 and 20 in the PAN neck, respectively. Specifically, the RFADown numbered 17 is located in the scale transformation stage from 80 × 80 to 40 × 40 and downsamples the feature map from 128 × 80 × 80 to 128 × 40 × 40. The RFADown numbered 20 is located in the scale transformation stage from 40 × 40 to 20 × 20 and downsamples the feature map from 256 × 40 × 40 to 256 × 20 × 20. After multi-scale feature fusion, the detection head receives three-scale feature maps with sizes of 128 × 80 × 80, 256 × 40 ×40 and 512 × 20 × 20 and outputs category prediction results and bounding box regression results for cow targets. Except for the above module replacements, the hierarchical structure, connection relations and detection head configuration of the remaining parts of the network are consistent with the baseline YOLOv10s.

#### 2.2.2. Improvement of Downsampling Modules

In high-density cow breeding scenarios, partial occlusion frequently occurs among cows, with only body regions such as the head, back or abdomen remaining visible. Traditional downsampling operations may lose such fine-grained features during spatial compression, which leads to missed detection of severely occluded or highly overlapping cow targets. To address this problem, this paper replaces the original SCDown module in the PAN neck network with the RFADown module. Each RFADown module consists of Receptive Field Attention Convolution (RFAConv) [30] and a dual-branch pooling structure. These two components work collaboratively to combine local feature retention with adaptive feature weighting within the receptive field during spatial compression, so as to enhance the feature representation capability of the model for densely distributed and partially occluded cows. In this module, RFAConv mainly combines Receptive Field Attention (RFA) with standard convolution operations.

**Figure 4:**
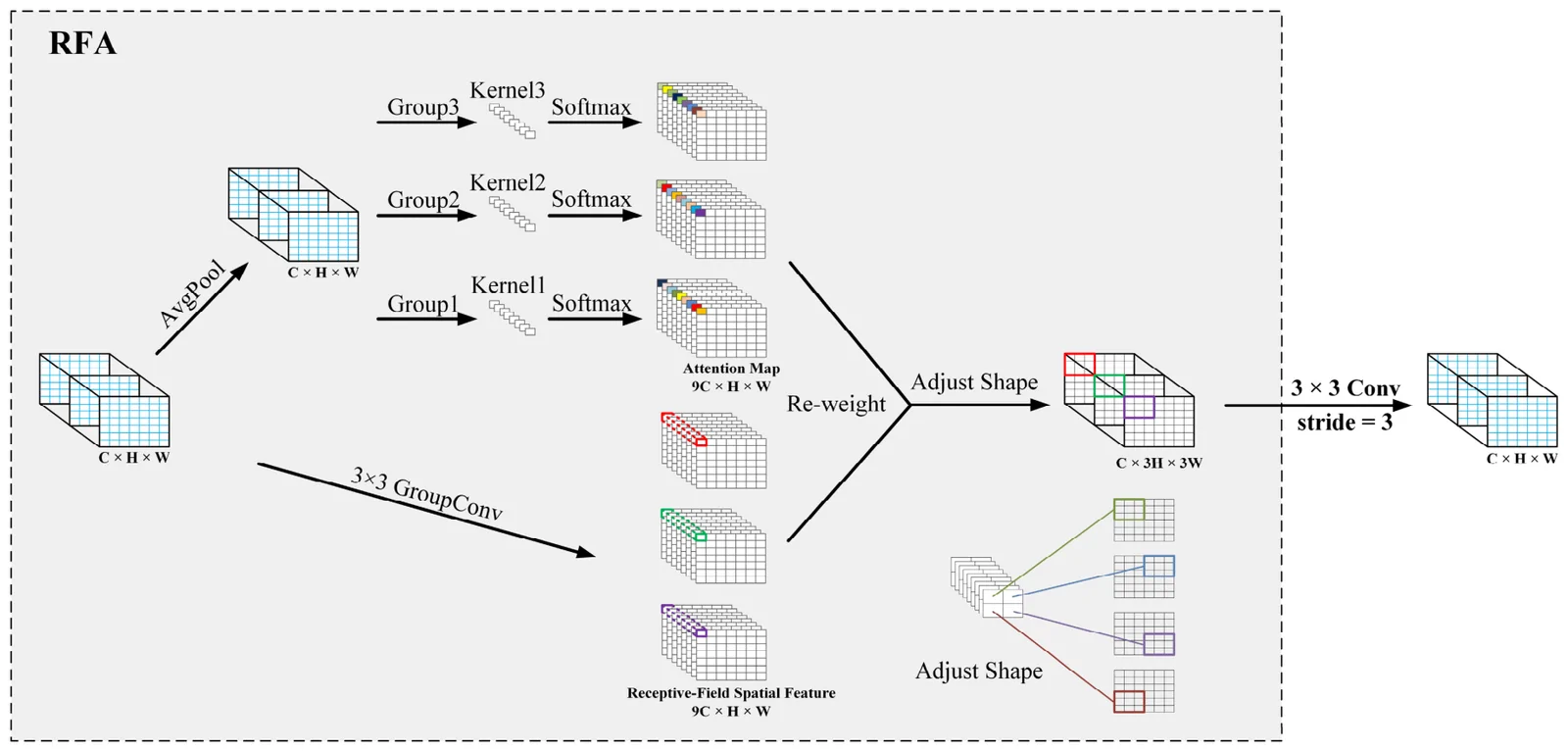
Receptive Field Attention (RFA) mechanism. Different colors distinguish feature elements and illustrate their correspondence during feature reshaping.

As illustrated in Figure 4, RFAConv employs a 3 × 3 convolutional kernel for feature extraction and utilizes average pooling to achieve global information aggregation within each receptive field. Subsequently, a 1 × 1 grouped convolutional operation is utilized to facilitate interaction between information fragments. Finally, softmax is applied to emphasize the importance of each feature within its respective receptive field. The computation of RFA is shown in Equations (1)–(3).

(1) $$A_{\mathrm{RFA}}={\mathrm{Softmax}}_{k^{2}}\left[\mathrm{Reshape}\left(g_{C\to Ck^{2}}^{1\times 1}\left({\mathrm{AvgPool}}_{k,s,p}(X)\right)\right)\right]$$

(2) $$F_{\mathrm{RFA}}=\mathrm{Reshape}\left[\mathrm{ReLU}\left({\mathrm{BN}}_{\gamma ,\beta }\left(g_{C\to Ck^{2}}^{k\times k}(X)\right)\right)\right]$$

(3) $$F=A_{\mathrm{RFA}}⊙F_{\mathrm{RFA}}$$

In the equations, $X\in {\mathbb{R}}^{B\times C\times H\times W}$ denotes the input feature map, where *B*, *C*, *H* and *W* represent the batch size, number of channels, feature-map height, and feature-map width, respectively. *k*, *s* and *p* denote the kernel size, stride, and padding, respectively. $g_{C\to Ck^{2}}^{1\times 1}$ and $g_{C\to Ck^{2}}^{k\times k}$ denote the 1 × 1 and *k* × *k* grouped convolutions that expand the number of channels from *C* to *Ck*^2^, respectively. Reshape denotes the rearrangement of the feature tensor from $B\times Ck^{2}\times H^{\prime }\times W^{\prime }$ to $B\times C\times k^{2}\times H^{\prime }\times W^{\prime }$. ${\mathrm{Softmax}}_{k^{2}}$ denotes normalization along the *k^2^* receptive-field positions to obtain the receptive-field attention weights *A_RFA_*. ${\mathrm{BN}}_{\gamma ,\beta }$ denotes batch normalization with learnable scaling parameter γ and bias parameter β, followed by ReLU activation and reshaping to obtain the receptive-field spatial features *F_RFA_*. The symbol ⊙ denotes element-wise multiplication, and *F* represents the attention-weighted receptive-field features. Here, *H*′ = [(*H* + 2*p* − *k*)/*s*] + 1 and *W*′ = [(*W* + 2*p* − *k*)/*s*] + 1 denote the height and width of the transformed feature map, respectively.

**Figure 5:**
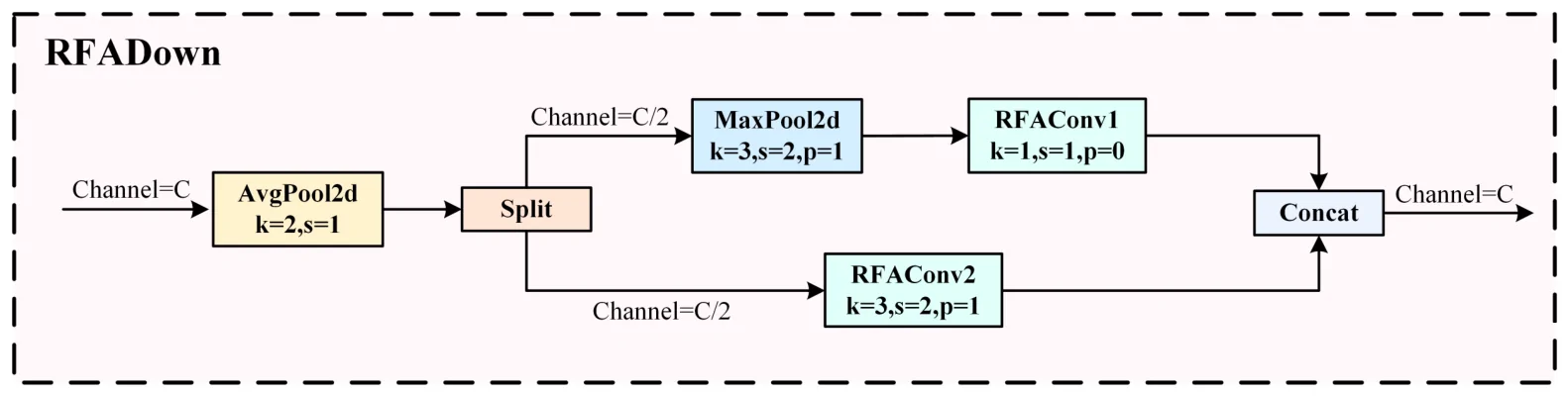
Structure diagram of RFADown module.

As shown in Figure 5, the RFADown module first preprocesses the input feature map with average pooling that has a 2 × 2 pooling kernel and a stride of 1. It then equally divides the obtained features into two branches along the channel dimension, with each branch containing half the number of input channels. The upper branch performs spatial downsampling using max pooling with a 3 × 3 pooling kernel, a stride of 2 and a padding of 1 to retain local features with strong responses, and then conducts feature mapping via RFAConv with a 1 × 1 convolution kernel and a stride of 1. The lower branch directly adopts RFAConv with a 3 × 3 convolution kernel, a stride of 2 and a padding of 1, which extracts receptive-field-aware spatial features while completing spatial downsampling. Finally, the outputs of the two branches are concatenated along the channel dimension to obtain a downsampled feature map with C channels. While accomplishing spatial downsampling, RFADown can preserve local high-response features of occluded cows and extract receptive field spatial information, thereby enhancing the model’s detection capability for densely distributed and partially occluded cow targets.

#### 2.2.3. Partial Bi-Level Routing Attention Module

In actual cow farm monitoring scenarios, differences in shooting distance and cow activity areas usually lead to non-uniform spatial distribution of cow targets. Dense aggregation and partial occlusion of targets frequently occur in local areas, which increases the difficulty of feature extraction and target distinction. To improve the detection performance of the network under such complex spatial distribution conditions, this paper retains the partial channel processing and feature fusion framework of Partial Self-Attention (PSA) and replaces the original self-attention unit therein with Bi-Level Routing Attention (BRA) to construct the PBRA module. BRA adopts a bi-level routing mechanism. It first evaluates the semantic correlation between query regions and candidate regions at the coarse-grained region level, and screens candidate regions with high correlation. Subsequently, fine-grained attention calculation is performed based on the selected regions. While reducing redundant attention calculation, this mechanism helps enhance the network’s feature aggregation capability for densely distributed and partially occluded cow targets.

**Figure 6:**
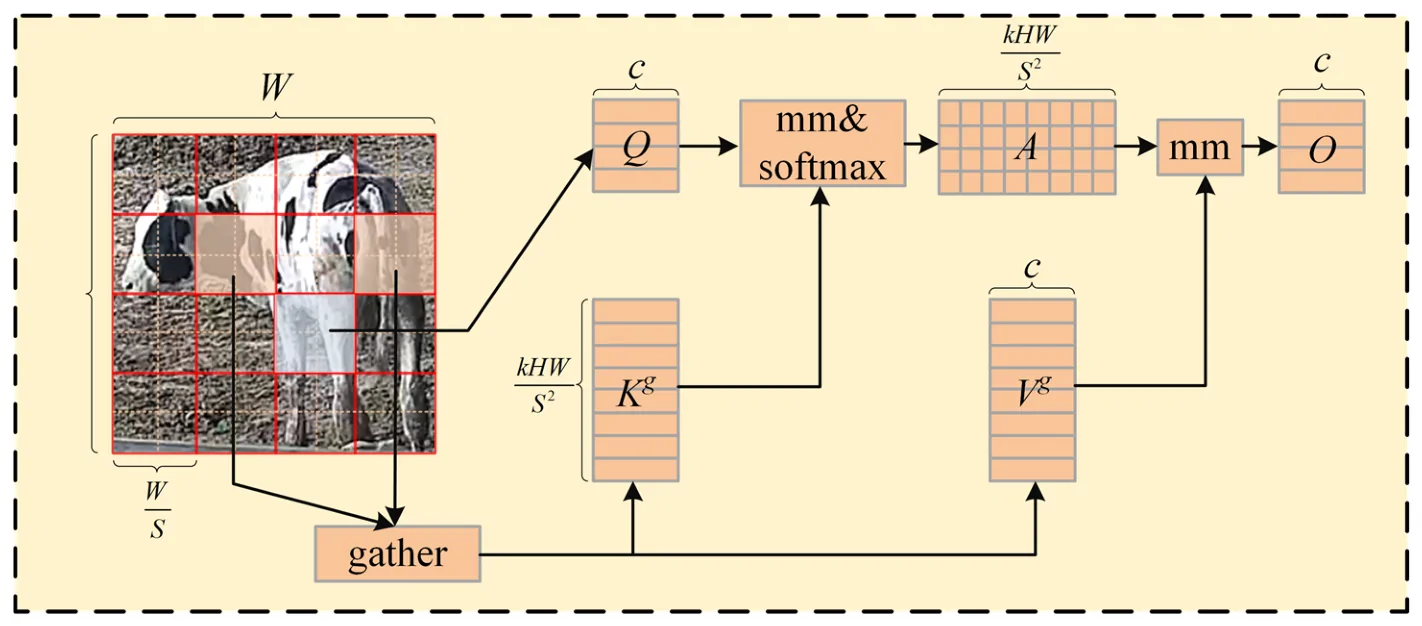
Feature aggregation in Bi-Level Routing Attention. (Key and value features (K^g^ and V^g^) are gathered from the top-k relevant regions. Query features Q and gathered keys K^g^ are used to compute attention weights A, which are applied to V^g^ to obtain the output O; “mm” denotes matrix multiplication).

The principle of BRA is illustrated in Figure 6. The feature map $X\in R^{H\times W\times C}$ is divided into S×S non-overlapping regions, with each region containing $\frac{HW}{S^{2}}$ feature vectors. Specifically, $X^{r}\in R^{S^{2}\times \frac{HW}{S^{2}}\times O}$, query, key, and value are set as $Q,K,V\in R^{S^{2}\times \frac{HW}{S^{2}}\times C}$. The adjacency matrix $A^{r}$, which evaluates the semantic relationships between regions, is calculated according to Equation (4).

(4) $$A^{r}=Q^{r}{\left(K^{r}\right)}^{T}$$

Then, the top-k indices with the highest regional relevance are identified to prune the association graph.

(5) $$I^{r}=TopkIndex(A^{r})$$

Utilizing the index matrix $I^{r}$, as shown in Equation (5), fine-grained token-to-token attention across regions is implemented by first collecting the key–value tensors $V^{g}=gather(V,I^{r})$ and $K^{g}=gather(K,I^{r})$. Subsequently, attention is focused on the collected key–value pairs, and a Local Context Enhancement (LCE) term is introduced, which is realized through depthwise separable convolutions.

**Figure 7:**
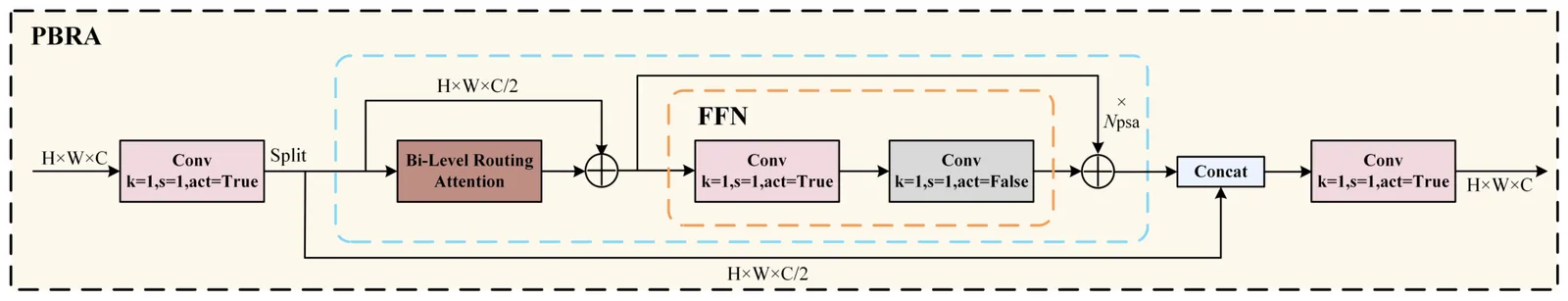
Structure of the PBRA module. Arrows indicate feature flow; ⊕ denotes element-wise addition.

The specific structure of the PBRA module is shown in Figure 7. For the input feature map $X\in R^{H\times W\times C}$, a 1 × 1 convolution is first applied for feature transformation, after which the resulting feature map is evenly split into two branches along the channel dimension, $X_{1},X_{2}\in R^{H\times W\times C/2}$. Among them, *X*_1_ is directly retained as a bypass feature, while *X*_2_ is first enhanced by BRA and then fused with its input through a residual connection. Subsequently, the feature is further transformed by the FFN and again fused through a residual connection. Throughout the above process, the feature size remains *H* × *W* × *C*/2. Finally, the enhanced *X*_2_ is concatenated with the bypass feature *X*_1_ along the channel dimension, restoring the feature size to *H* × *W* × *C*, and a 1 × 1 convolution is then applied to obtain the fused feature map.

#### 2.2.4. Improvement of Spatial Pyramid Pooling

**Figure 8:**
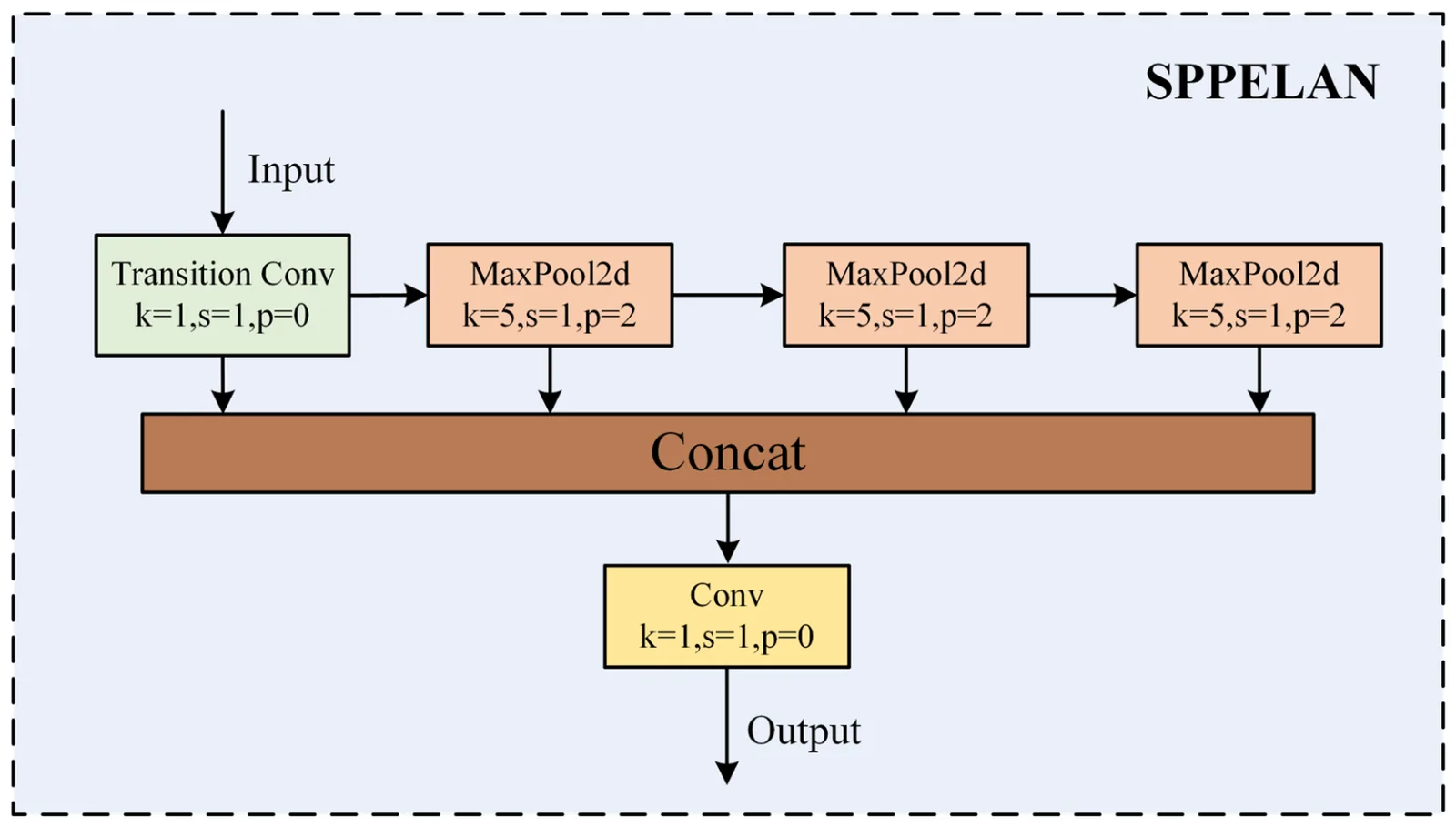
Structure of the SPPELAN module.

The specific structure of the SPPELAN module is shown in Figure 8. The input feature map first undergoes channel mapping through a 1 × 1 transition convolution to obtain initial features, and then sequentially passes through three max pooling layers with a pooling kernel size of 5 × 5, a stride of 1 and a padding of 2. Since each pooling layer takes the output of the previous stage as its input, the effective receptive field of features expands step by step along with the pooling process, while the spatial resolution remains unchanged. The initial convolutional features and the outputs of the three pooling stages are concatenated along the channel dimension. Finally, channel integration is completed via 1 × 1 convolution to obtain the output features of the SPPELAN module. By aggregating context information within different receptive field ranges, this module helps enhance the network’s feature representation capability for cow targets of different scales.

### 2.3. Multi-Object Tracking Method

#### 2.3.1. Dairy Cow Multi-Object Tracking Framework

**Figure 9:**
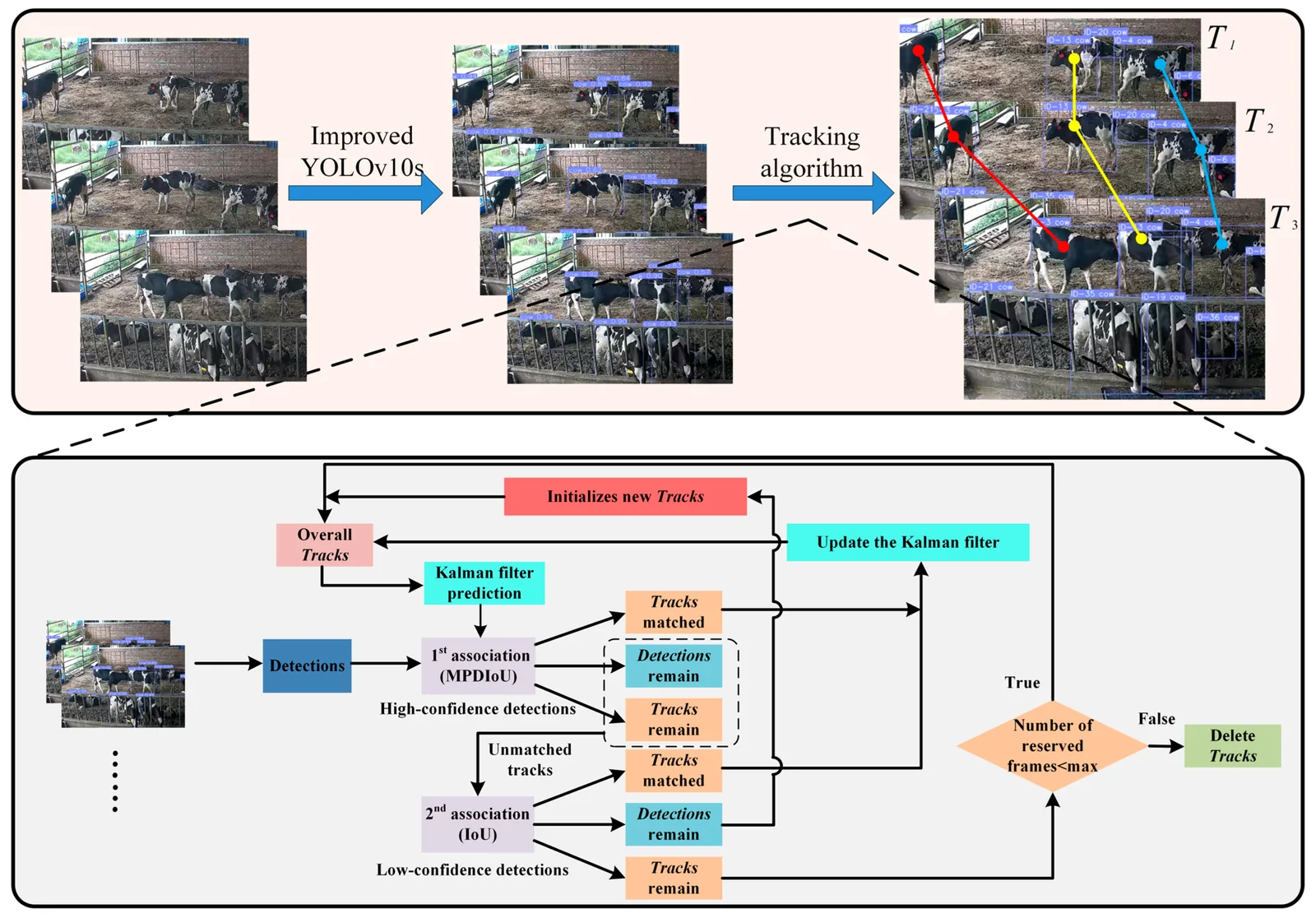
Workflow of the dairy cow multi-object tracking algorithm.

The workflow of the tracking algorithm adopted in this paper is shown in Figure 9. The improved YOLOv10s detector is used to detect cow targets in each frame, with the detection confidence threshold set to 0.30. Specifically, the retained bounding boxes are further divided into high-confidence bounding boxes (s ≥ 0.50) and low-confidence bounding boxes (0.30 ≤ s < 0.50). In subsequent frames, the Kalman filter is adopted to predict the motion states of existing trajectories. The Kalman filter uses an 8-dimensional constant velocity motion state vector (*x*, *y*, *a*, *h*, *v_x_*, *v_y_*, *v_a_*, *v_h_*), where (*x*, *y*), *a*, and *h* represent the center coordinates, aspect ratio, and height of the target bounding box respectively, and the last four terms represent the corresponding velocity components. The time step is set to 1, and the standard deviation weights for position and velocity are set to 1/20 and 1/160 respectively.

After obtaining the predicted states of existing trajectories, MPDIoU is first applied to associate high-confidence bounding boxes with predicted trajectories, with the matching threshold set to 0.90. For trajectories remaining unmatched after the first association, IoU is further used for a second association with low-confidence bounding boxes, with the matching threshold set to 0.50. After the completion of the above two-stage association, unmatched bounding boxes with confidence no less than 0.60 are used to initialize new trajectories. For new trajectories initialized by unmatched bounding boxes in frames after the first frame, they are first marked as unconfirmed trajectories. Their state is updated to confirmed only when the trajectory is successfully re-associated in the next frame with a matching threshold of 0.70; otherwise, they are removed. Confirmed trajectories that fail to be matched are marked as lost and retained in the trajectory buffer for a maximum of 210 frames. Since the frame rate of the videos used in this study is 30 FPS, this setting corresponds to a maximum trajectory retention duration of approximately 7 s. If a lost trajectory cannot be re-associated within this retention duration, it will be deleted upon reaching the maximum buffer length.

#### 2.3.2. Optimization of Trajectory Association

**Figure 10:**
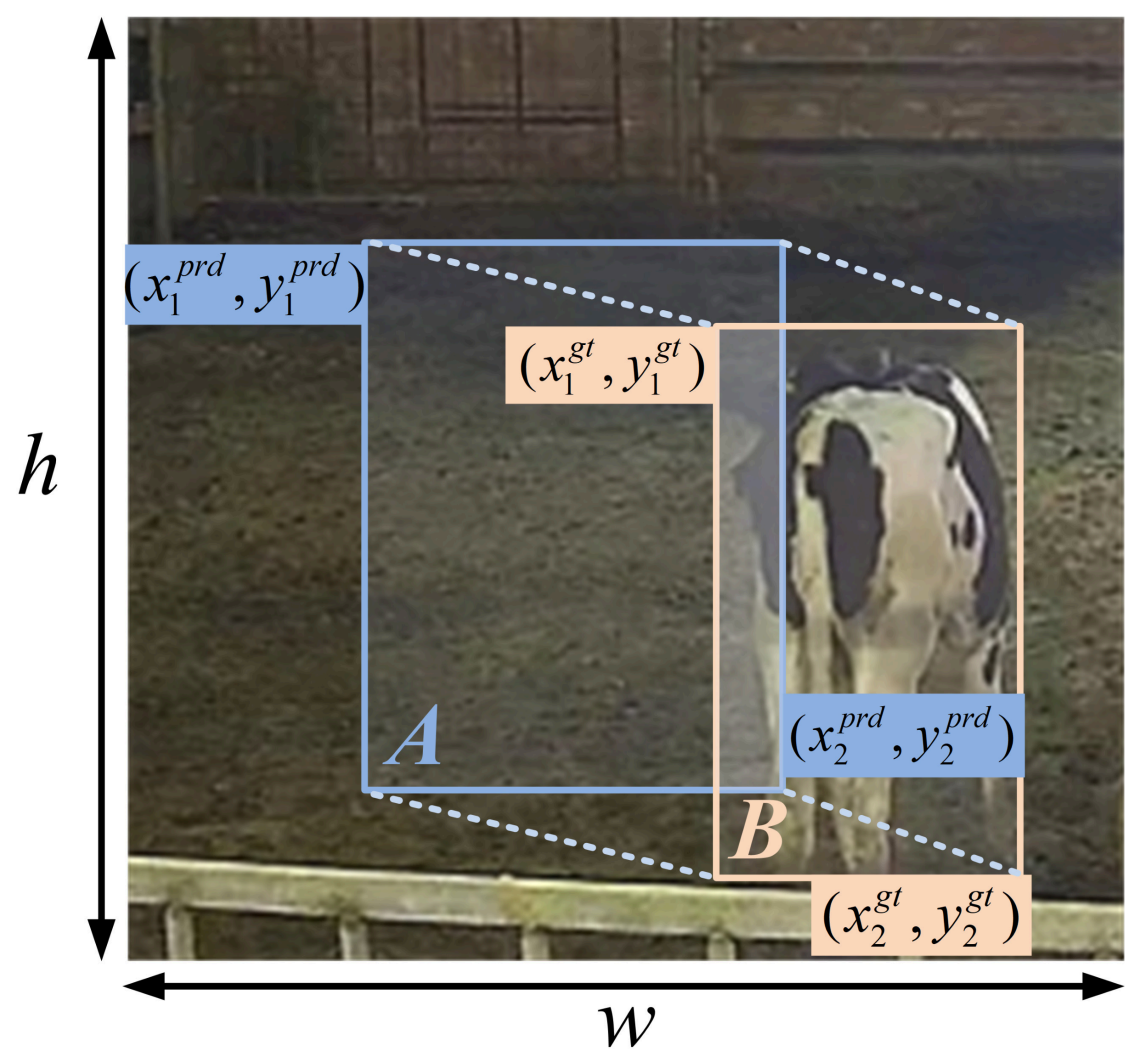
Geometric illustration of minimum point distance intersection over union (MPDIoU). (The blue box, A, and orange box, B, represent the predicted and detected bounding boxes, respectively. Dashed lines connect their corresponding top-left and bottom-right corners to calculate distance penalties; w and h denote the input-image width and height).

In multi-object tracking algorithms, Intersection over Union (IoU) association is a crucial step. It is used to evaluate the overlap between detection boxes and prediction boxes, and trajectory matching is performed based on this evaluation result. In actual dairy farm scenarios, cows are at different distances from the camera, and there are occlusions, overlaps, and uneven distributions among them. As a result, the aspect ratios and sizes of detection boxes vary significantly. Under such conditions, traditional IoU association methods have difficulty in accurately distinguishing between different cows. Moreover, when there is no overlapping area between a detection box and a prediction box, reliable gradient information cannot be calculated, which further affects the stability and accuracy of tracking. To address the association errors caused by traditional IoU-based matching, this study adopts the MPDIoU metric for initial association. Unlike conventional IoU methods that rely solely on overlap areas, MPDIoU additionally considers the geometric distances between corresponding corner points of bounding boxes, thereby improving association reliability under occlusion and overlap conditions, as illustrated in Figure 10 and Equations (6) and (7).

(6) $$IoU=\frac{A\cap B}{A\cup B}$$

(7) $$MPDIoU=IoU-\frac{{\left(x_{1}^{prd}-x_{1}^{gt}\right)}^{2}+{\left(y_{1}^{prd}-y_{1}^{gt}\right)}^{2}}{w^{2}+h^{2}}-\frac{{\left(x_{2}^{prd}-x_{2}^{gt}\right)}^{2}+{\left(y_{2}^{prd}-y_{2}^{gt}\right)}^{2}}{w^{2}+h^{2}}$$

where $\left(x_{1}^{gt},y_{1}^{gt}\right)$ and $\left(x_{2}^{gt},y_{2}^{gt}\right)$ are the coordinates of the upper-left and lower-right corners of the detection box, respectively, and $\left(x_{1}^{prd},y_{1}^{prd}\right)$ and $\left(x_{2}^{prd},y_{2}^{prd}\right)$ are the coordinates of the upper-left and lower-right corners of the prediction box, respectively. w and h represent the width and height of the input image. This global normalization ensures a uniform distance penalty scale for cow targets of different sizes, making the matching threshold equally applicable to both near and far individuals and avoiding scale bias in trajectory association.

For detection boxes and trajectories that remain unmatched after the initial matching step, a second association is performed based on IoU. Notably, in scenarios where target bounding boxes have inconsistent shapes or overlap with one another, MPDIoU can better capture the similarity between bounding boxes by considering the distances between the top-left and bottom-right points of each bounding box. This two-stage association strategy, combined with the advantages of MPDIoU, is designed to improve the robustness and reliability of trajectory matching under occlusion and overlap conditions while maintaining efficient tracking performance in complex dairy farm environments.

## 3. Experiments and Results

### 3.1. Experimental Platform and Parameter Settings

To investigate the tracking performance of the proposed method for cows in natural scenes, two types of experiments were conducted: (1) the Object detection experiment of dairy cows to verify the performance of the improved YOLOv10 model and (2) the multi-object tracking experiment on dairy cows to verify the performance of the BR-tracker. The experimental platform was a 64-bit Windows 10 operating system, with hardware configurations including an Intel(R) Core(TM) i5-14600KF 3.5 GHz processor, an NVIDIA GeForce RTX 4060Ti with 16 GB of memory, and 32 GB of RAM. CUDA version 11.8, Python version 3.9, and PyTorch version 2.0.1 were used for the experiments.

To ensure a fair comparison among different detection models, all models were trained using the same dataset split and main training hyperparameters. The input image size was set to 640 × 640 pixels, the batch size was 16, and the number of training epochs was 200. AdamW was used as the optimizer, with the momentum parameter and weight decay set to 0.937 and 0.0005, respectively. The initial learning rate was set to 0.01, and a linear learning-rate decay strategy with three warm-up epochs was adopted. Mosaic augmentation, horizontal flipping, random scaling, and HSV color-space augmentation were applied during training, with the corresponding parameters set to mosaic = 1.0, fliplr = 0.5, scale = 0.5, *hsv_h* = 0.015, *hsv_s* = 0.7, and *hsv_v* = 0.4. Mosaic augmentation was disabled during the final 10 training epochs. All models were initialized using their corresponding pretrained weights to accelerate convergence. To clearly display the experimental settings and facilitate independent reproduction by other researchers, the main training parameters and tracking parameters adopted in this paper are summarized in Table A1 of Appendix A.

### 3.2. Evaluation Metrics

#### 3.2.1. Object Detection Evaluation Metrics

In the task of object detection, evaluation metrics serve as pivotal factors for assessing model performance. This study utilizes seven metrics to evaluate the performance of cow detection models, as outlined below: P (precision) represents the proportion of correctly predicted positive cow instances; R (recall) indicates the proportion of actual positive cow instances; mAP (mean average precision) denotes the mean of average precision values; GFLOPs is a metric used to quantify the computational complexity of the model; Params refers to the total number of learnable parameters in the neural network, including weights (Z) and biases (D); FPS stands for Frames Per Second. The definitions of these metrics are provided through Equations (8)–(12).

(8) $$P=\frac{TP}{TP+FP}\times 100\%$$

(9) $$R=\frac{TP}{TP+FN}\times 100\%$$

(10) $$mAP=\frac{\sum_{c=1}^{C}\int_{0}^{1}P_{c}\left(R\right)dR}{C}$$

(11) Params=(Z+D)

(12) $$FPS=\frac{1}{t}$$

In this context, TP (true positives) represents the number of positive samples correctly identified; FN (false negatives) indicates the cases where the model incorrectly predicts positive samples as negative; FP (false positives) signifies the instances where the model mistakenly classifies negative samples as positive. mAP (mean average precision) is the average of the average precision (AP) obtained when detecting dairy cows.

#### 3.2.2. Multi-Object Tracking Evaluation Metrics

This study adopts six primary evaluation metrics to compare the performance of multi-object tracking in cows, namely Identification F1 (IDF1), ID switch (IDS), Multi-Object Tracking Accuracy (MOTA), Multi-Object Tracking Precision (MOTP), Higher-Order Tracking Accuracy (HOTA) [31] and average Frames Per Second (FPS). IDF1 assesses the stability of the tracking algorithm. MOTA measures the performance of the tracking algorithm in detecting targets and maintaining their trajectories. MOTP reflects the accuracy of tracking performance, quantifying the localization precision of the detector. HOTA is a high-dimensional tracking accuracy metric that provides a balanced consideration of various performance aspects in a single indicator.

IDF1 is calculated as shown in Equation (13). Where IDTP represents the total number of targets correctly tracked without changing their IDS; IDFP represents the total number of targets incorrectly tracked without changing their IDS; and IDFN represents the total number of targets lost during tracking without changing their IDS.

(13) $$IDF1=\frac{2IDTP}{2IDTP+IDFP+IDFN}$$

MOTA is calculated as shown in Equation (14), where FP represents the number of false positives at time t, FN represents the number of false negatives at time t, IDS represents the number of ID switches at frame t, and $g_{t}$ represents the total number of all objects.

(14) $$MOTA=1-\frac{\sum_{t}\left(FP+FN+IDS\right)}{\sum_{t}g_{t}}$$

MOTP is calculated as shown in Equation (15), where $d_{i,t}$ represents the distance between a given object and its paired hypothetical position in frame t; $c_{t}$ represents the number of matches between targets and hypothetical positions in frame t, and i represents the current detected target.

(15) $$MOTP=\frac{\sum_{t,i}d_{i,t}}{\sum_{t}c_{t}}\times 100\%$$

HOTA is calculated as shown in Equation (16), where DetA represents the detection accuracy score, and AssA represents the association accuracy score. A(c) represents the association accuracy, and C is a point belonging to TP. TP is the number of positive samples; FN is the number of positive samples predicted as negative by the model; FP is the number of negative samples predicted as positive by the model.

(16) $$HOTA=\sqrt{DetA\cdot AssA}=\sqrt{\frac{\sum_{C\in TP}A\left(c\right)}{TP+FN+FP}}$$

In addition, IDS and FPS are also used for evaluation in this study. Higher values of IDF1, MOTA, MOTP, HOTA, and FPS, along with lower values of IDS, indicate better model performance.

### 3.3. Detection Results for Densely Populated Dairy Cows

#### 3.3.1. Comparison of Different Detection Models

To evaluate the detection performance of the improved YOLOv10s model, this study carries out comparative experiments on YOLOv5s, YOLOv8s, the original YOLOv10s, YOLOv5s-CA proposed by Guo et al. [23], and Pruned YOLO proposed by Zheng et al. [27]. All models are trained and evaluated with identical dataset partitions and experimental configurations. Five different random seeds are assigned to each model for independent repeated trials, and experimental results are reported in the form of mean ± standard deviation. As shown in Table 1, the BR-YOLOv10s proposed in this paper achieves the best overall detection performance, with P, R and mAP reaching 95.7 ± 0.3%, 90.3 ± 0.2% and 95.2 ± 0.2% respectively. Compared with YOLOv5s-CA, P, R and mAP are increased by 1.9, 2.1 and 2.0 percentage points respectively. In addition, both P and R of BR-YOLOv10s are improved by 4.6 percentage points and mAP is improved by 3.8 percentage points compared with Pruned YOLO, which indicates that it has stronger detection capability in scenarios with densely distributed cows. Although the parameter count of BR-YOLOv10s rises by 0.47 M and GFLOPs increase by 2.3 compared with the original YOLOv10s, its P, R and mAP are boosted by 2.3, 1.9 and 1.3 percentage points respectively. This result reveals that the proposed model obtains better detection performance with a limited growth in model complexity. When compared with YOLOv8s, BR-YOLOv10s raises mAP by 1.5 percentage points while reducing parameters by 3.44 M and cutting computational cost by 4.7 GFLOPs. The small standard deviations obtained from five independent repeated trials also demonstrate that the proposed model delivers relatively stable detection performance. Overall, BR-YOLOv10s achieves a favorable balance among detection accuracy, model complexity and experimental stability, and it is more suitable for cow detection under dense distribution and partial occlusion conditions.

**Figure 11:**
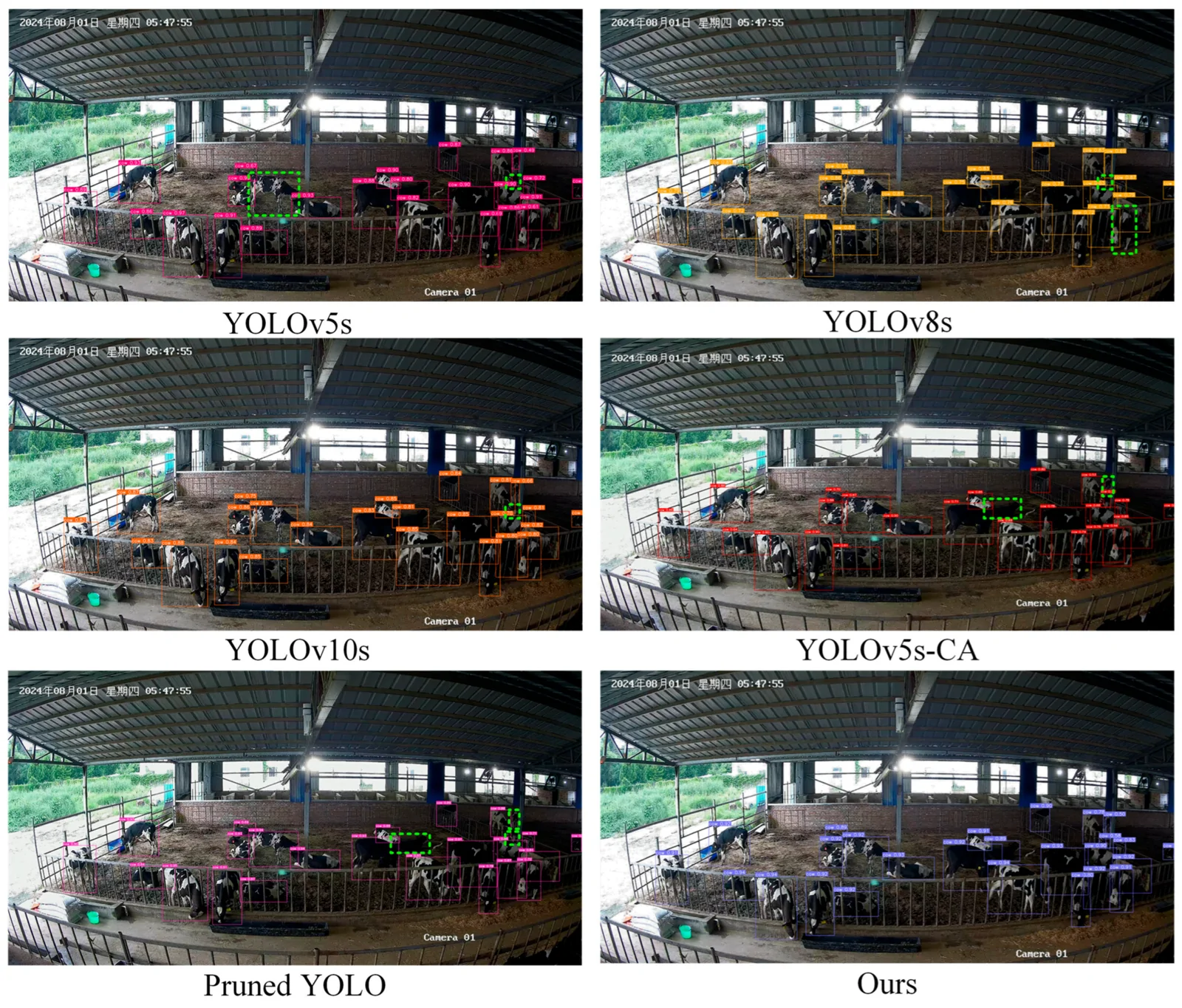
Comparison of detection results obtained using different algorithms. Missed targets are indicated by green dashed boxes. (The Chinese timestamp labels “年”, “月”, “日”, and “星期” denote the year, month, day, and day of the week, respectively).

Furthermore, as shown in the visualization results of different detection algorithms in Figure 11, YOLOv5s and YOLOv8s exhibited missed detections under partial occlusion conditions, with two missed cows for each model. Although YOLOv10s improved the detection performance, it still failed to detect one small cow located on the right side of the image. By introducing an attention mechanism, YOLOv5s-CA improved the detection performance to a certain extent; however, two cows were still missed in densely occluded regions. Due to the loss of feature information caused by model pruning, Pruned YOLO missed three cows in complex scenarios. In contrast, the proposed BR-YOLOv10s successfully detected all visible cows and effectively reduced missed detections in densely occluded scenes. These quantitative results further indicate that the proposed model exhibits stronger detection robustness and localization performance in densely distributed and partially occluded dairy cow scenarios.

In conclusion, through comprehensive experimental comparisons among the six detection models, the proposed BR-YOLOv10s achieves the best overall performance, particularly in densely distributed and partially occluded dairy cow scenarios, where missed detections were effectively reduced.

#### 3.3.2. Heatmap Analysis

**Figure 12:**
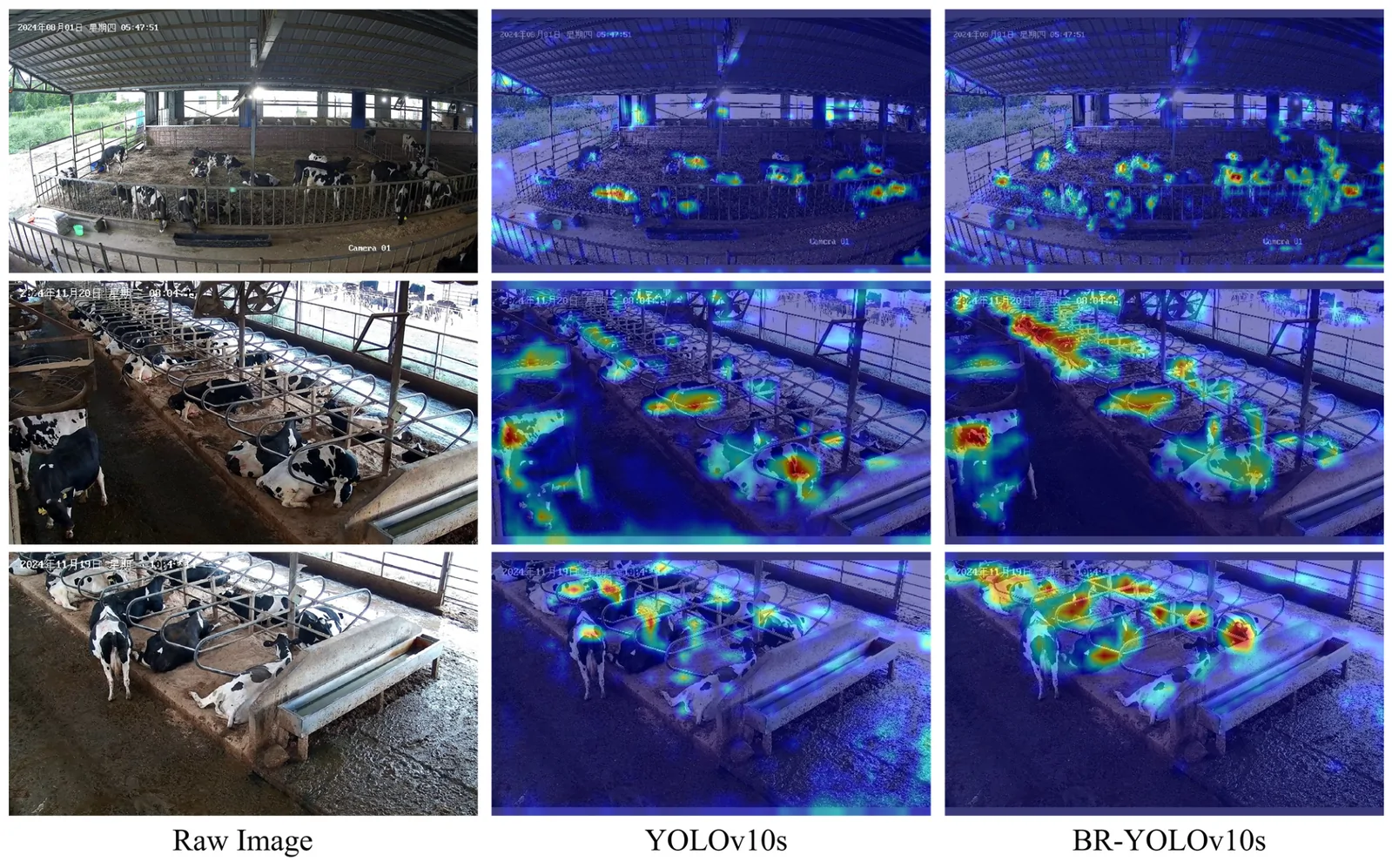
Comparative analysis of heatmaps before and after model improvement. (The Chinese timestamp labels “年”, “月”, “日”, and “星期” denote the year, month, day, and day of the week, respectively).

To further validate the effectiveness of the proposed improvements, Grad-CAM++ [32] was employed to visualize the heatmaps of the object detection results, where red regions indicate higher attention weights and blue regions indicate lower attention weights. As shown in Figure 12, compared with the original YOLOv10s model, the improved BR-YOLOv10s model exhibits more concentrated and comprehensive attention toward cow targets. The high-response regions cover the main body of the cows more completely, while interference from background regions is significantly reduced. In addition, the boundaries of the attention regions are clearer and more distinct, indicating that the proposed model can more accurately distinguish cow targets from background information in complex barn environments. Overall, the heatmap analysis further demonstrates the effectiveness of the proposed method in complex scenarios. The visualization results indicate that the improved model is able to focus more on the main body regions of cow targets while avoiding excessive responses to irrelevant background information. This demonstrates that the model possesses stronger target feature extraction and target representation capabilities, while also enhancing the interpretability of the detection results.

#### 3.3.3. Ablation Experiments and Attention Module Comparisons

To evaluate the effectiveness of each improved module for dense dairy cow detection in intensive farming scenarios and to analyze the applicability of different feature selection mechanisms in the proposed network, this study used YOLOv10s as the baseline and conducted module ablation experiments and comparative experiments with different attention modules. The results are shown in Table 2. Among them, Groups 1, 2, 3, and 5 retained the original PSA module in YOLOv10s; Groups 4, 6, 7, and 11 used PBRA to replace PSA; and Groups 8 to 10 used BRA, SK, and CBAM to replace PSA, respectively. All attention modules were placed at the original position of PSA, while the configurations of RFADown and SPPELAN were kept unchanged, so as to compare the detection performance and computational cost of different module configurations. Each model was independently repeated using five different random seeds, and the experimental results are reported as mean ± standard deviation.

In the single-module configurations, after introducing RFADown, the R and mAP of the model increased from 88.4 ± 0.3% and 93.9 ± 0.3% to 88.7 ± 0.5% and 94.4 ± 0.4%, respectively, whereas P decreased from 93.4 ± 0.4% to 92.2 ± 0.6%. RFADown adopts a dual-branch downsampling structure and a receptive field attention mechanism to enhance the preservation and representation of locally visible features of partially occluded dairy cows during downsampling, which helps reduce missed detections of such targets. However, in regions where dairy cows are densely distributed and target boundaries overlap, the similarity between background textures and local features of adjacent cows may cause some non-target regions to be incorrectly identified, resulting in a decrease in P. When SPPELAN was introduced alone, P decreased slightly from 93.4 ± 0.4% to 93.2 ± 0.4%. This change may be related to the fusion of information from adjacent cows and the background during multi-scale feature aggregation, which may interfere with the discrimination of some target features. When PBRA was introduced alone, P and mAP reached 94.6 ± 0.5% and 94.6 ± 0.2%, respectively, which were the highest values among the three single-module configurations, indicating that this structure achieved good detection performance under the current experimental conditions. In the dual-module configurations, after further introducing SPPELAN on the basis of RFADown, P increased from 92.2 ± 0.6% to 93.5 ± 0.5%, and R increased from 88.7 ± 0.5% to 89.8 ± 0.5%. This indicates that the multi-scale contextual information provided by SPPELAN can complement the local features preserved by RFADown, thereby enhancing the joint representation of local features and contextual information. After further introducing RFADown on the basis of PBRA, P decreased slightly from 94.6 ± 0.5% to 94.4 ± 0.4%. This change may be related to the fact that, while RFADown enhances locally visible features, it also preserves some similar feature responses from adjacent cows and target overlapping regions. The combination of SPPELAN and PBRA achieved an mAP of 95.0 ± 0.2%, which was the highest among the dual-module configurations.

In the attention module comparison involving Groups 5 and 8 to 11, RFADown and SPPELAN were kept unchanged, while PSA, BRA, SK, CBAM, and PBRA were respectively used at the original position of PSA to compare the effects of different attention modules on detection performance and computational cost. When the original PSA was retained, the mAP of the model was 94.6 ± 0.4%. After replacing it with BRA, SK, and CBAM, respectively, the mAP decreased to 93.5 ± 0.6%, 92.0 ± 0.6%, and 91.6 ± 0.8%, respectively. When PBRA was used, the mAP increased by 0.6 percentage points compared with the original PSA. Compared with the configuration using the BRA module, PBRA embeds region-routing attention into the branch that processes partial channels and fuses it with the bypass features, resulting in improvements of 1.9, 1.4, and 1.7 percentage points in P, R, and mAP, respectively. These results indicate that different selection mechanisms differ in their applicability under the current network configuration, and PBRA achieved the highest detection accuracy among the compared models. In terms of model complexity, the model using the PBRA module had 7.70 M parameters and 23.9 GFLOPs. Compared with the model using the BRA module, its parameter count and computational cost increased by 0.05 M and 0.4 GFLOPs, respectively, while achieving higher detection accuracy. Compared with the model using the SK module, the two metrics decreased by 0.04 M and 0.7 GFLOPs, respectively, while mAP increased by 3.2 percentage points. The model using the CBAM module had a lower parameter count and computational cost, at 6.65 M and 22.8 GFLOPs, respectively, but its mAP was 3.6 percentage points lower than that of the model using the PBRA module. This indicates that the PBRA model achieved higher detection accuracy than the CBAM model at the cost of a certain increase in computational overhead.

When RFADown, SPPELAN, and PBRA were introduced simultaneously, the model achieved the best performance, with P, R, and mAP reaching 95.7 ± 0.3%, 90.3 ± 0.2%, and 95.2 ± 0.2%, respectively. Compared with the baseline YOLOv10s, the three metrics increased by 2.3, 1.9, and 1.3 percentage points, respectively, while the parameter count and computational cost increased by 0.47 M and 2.3 GFLOPs, respectively. These results indicate that the three improved modules enhance the detection performance of the model from the perspectives of local feature preservation, multi-scale information aggregation, and target feature representation, respectively, enabling BR-YOLOv10s to achieve a good balance between detection accuracy and model complexity in scenarios with densely distributed and partially occluded dairy cows.

### 3.4. Multi-Object Tracking of Densely Populated Dairy Cows

#### 3.4.1. Comparison of Tracking Performance Before and After Algorithm Improvement

To verify the overall tracking performance of the proposed BR-Tracker and analyze the contributions of the detector and tracker improvements, this study randomly selects 40 videos from the original MOT dataset containing 130 videos to conduct standard MOT evaluation. The selected videos cover different monitoring positions under daytime and nighttime conditions, and each video contains approximately 20 cows. All model combinations are evaluated on the same 40 video sequences, and the evaluation metrics include IDF1, IDS, MOTA, MOTP, HOTA and FPS.

As shown in Table 3, this study combines different detection models with BR-Tracker to evaluate their impact on tracking performance. While keeping the dataset partition, training settings and experimental conditions consistent, each detector configuration is independently trained with five different random seeds. The detector weights obtained from each training run are used to perform tracking evaluation on all 40 video sequences, and the tracking accuracy metrics are reported as the mean ± standard deviation of five experiments. Among the evaluated model combinations, YOLOv5s+BR-Tracker achieves the highest processing speed of 48.9 FPS, but its IDF1 and HOTA are 68.9 ± 1.2% and 58.9 ± 0.9%, respectively, indicating that this combination has a relatively limited cow identity association capability in dense occlusion scenarios. After introducing the coordinate attention mechanism, the IDF1 and HOTA of YOLOv5s-CA+BR-Tracker increase to 70.6 ± 0.8% and 60.4 ± 1.0% respectively, while the IDS decreases from 1828.6 ± 75.6 to 1745.2 ± 76.2, indicating that strengthening the feature expression of key regions helps improve target association capability. The IDF1 and HOTA of Pruned YOLO+BR-Tracker reach 73.4 ± 1.1% and 61.9 ± 1.2% respectively, with a processing speed of 44.2 FPS, demonstrating that the pruned model still possesses good real-time tracking capability. Among all compared baseline detectors, YOLOv10s+BR-Tracker achieves the best overall tracking accuracy, with IDF1, MOTA and HOTA reaching 75.8 ± 0.7%, 78.5 ± 0.8% and 63.8 ± 0.9% respectively, and an IDS of 1587.6 ± 61.2. In comparison, BR-YOLOv10s+BR-Tracker achieves the highest IDF1, MOTA, MOTP and HOTA, reaching 79.2 ± 0.4%, 80.5 ± 0.7%, 79.1 ± 0.5% and 67.6 ± 0.6% respectively, while achieving the lowest IDS of 1446.4 ± 32.2. Compared with YOLOv10s+BR-Tracker, the above four accuracy metrics increase by 3.4, 2.0, 1.0 and 3.8 percentage points respectively; the IDS decreases by 8.89%; and the processing speed decreases from 44.7 FPS to 43.2 FPS. Although the improved modules introduce certain additional computational overhead, 43.6 FPS is still higher than the 30 FPS capture frame rate of the experimental videos and can meet the real-time processing requirement of single-channel surveillance video on the current experimental platform, indicating that the proposed algorithm achieves a good balance between tracking accuracy and computational efficiency.

To systematically verify the respective contributions of BR-YOLOv10s and BR-Tracker to the overall tracking performance, this study uses the combination of YOLOv10s and the ByteTrack [33] algorithm as the baseline to conduct ablation experiments, and the results are shown in Table 4. While keeping ByteTrack unchanged, after replacing YOLOv10s with BR-YOLOv10s, IDF1, MOTA, MOTP and HOTA increase by 3.6, 2.3, 1.2 and 1.8 percentage points respectively, and the IDS decreases from 1883.0 ± 76.2 to 1767.2 ± 50.8. While keeping the YOLOv10s detector unchanged, after replacing ByteTrack with BR-Tracker, IDF1, MOTA, MOTP and HOTA increase by 2.7, 3.7, 2.0 and 0.6 percentage points respectively, and the IDS decreases by 15.69%, indicating that the tracking algorithm proposed in this paper can improve the stability of trajectory association. When both BR-YOLOv10s and BR-Tracker are adopted simultaneously, the model achieves the optimal overall tracking performance. Compared with the baseline combination, its IDF1, MOTA, MOTP and HOTA increase by 6.1, 5.7, 3.0 and 4.4 percentage points respectively, and the IDS decreases by 23.19%.

**Figure 13:**
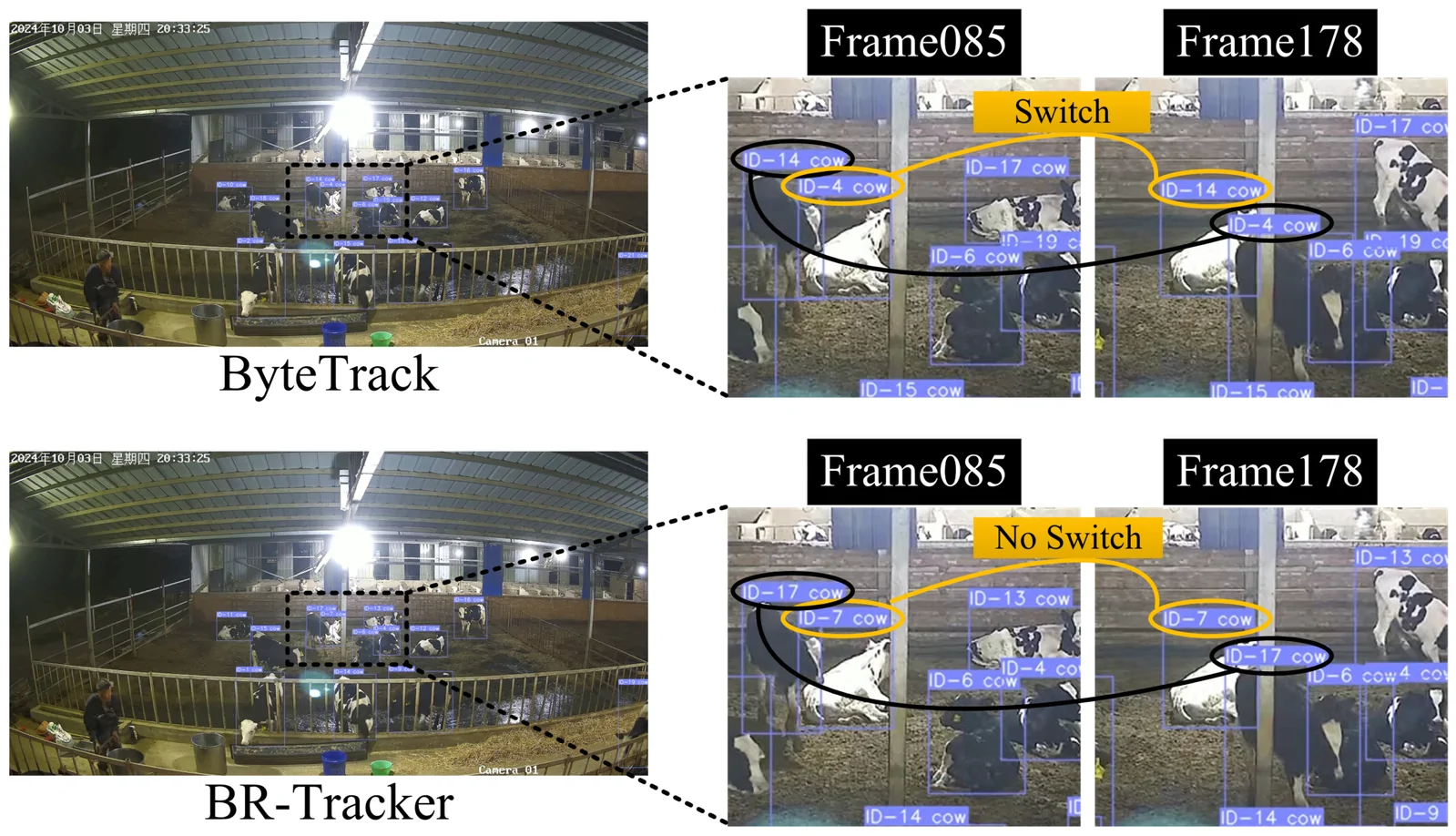
Comparison of tracking results before and after the proposed improvement. Black and orange circles indicate two individual cows, respectively, with each color identifying the same cow across frames. (The Chinese timestamp labels “年”, “月”, “日”, and “星期” denote the year, month, day, and day of the week, respectively).

The experimental details and results are illustrated in Figure 13. By observing the same video clip instance, it is found that after the cow in the black circle passes the cow in the orange circle, the IDs of the two cows using the ByteTrack algorithm are swapped, whereas no ID switch occurs when using the BR-Tracker. This demonstrates that the proposed method can effectively mitigate the issue of ID switch when tracking dairy cows in intensive farming scenarios, enabling it to better accomplish continuous tracking tasks.

#### 3.4.2. Comparison of Tracking Performance Across Representative Monitoring Scenarios

**Figure 14:**
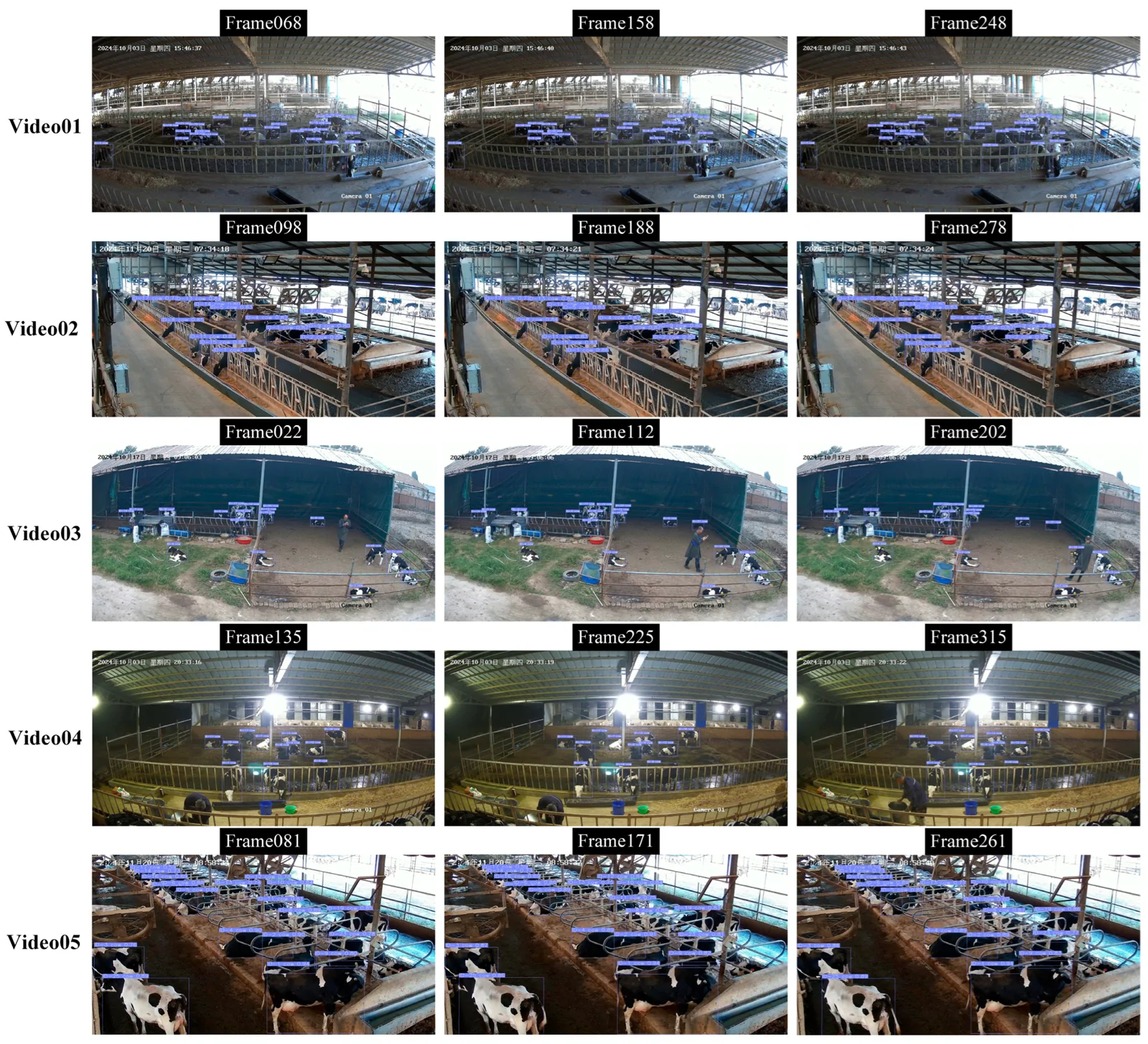
Tracking results of BR-Tracker under five representative monitoring scenarios. (The Chinese timestamp labels “年”, “月”, “日”, and “星期” denote the year, month, day, and day of the week, respectively).

To further analyze the performance differences of BR-Tracker across different typical monitoring scenarios, this study selects five representative videos from the 40 evaluation videos, covering different monitoring viewpoints, illumination conditions, spatial distribution of cows and degrees of target occlusion. Figure 14 presents the visualized tracking results of these five videos, and each scenario contains three temporal frames selected from the continuous video sequence at 3 s intervals.

The tracking performance of BR-Tracker under five typical monitoring scenarios is shown in Table 5. Among the five typical monitoring scenarios, video 03 achieves the best overall tracking performance, with MOTA, MOTP and HOTA reaching 85.9%, 83.7% and 73.4% respectively, while its IDS is the lowest, at only 9. As can be seen from Figure 13, the cows in this scenario are relatively sparsely distributed, the overlap degree between adjacent targets is low, and the daytime illumination is relatively stable, with relatively clear target boundaries. These conditions reduce matching ambiguity in the processes of object detection and data association, which is conducive to improving the stability of bounding box localization and maintaining the consistency of cow identities.

Video 01 and video 02 are also collected under daytime conditions, but their spatial distribution of cows differs significantly from that of video 03. In video 01, multiple cows are concentrated in the same fenced area, and there is a certain degree of occlusion between adjacent cows as well as between cows and foreground railings. Although the MOTA and MOTP of this video still reach 84.1% and 80.5% respectively, its HOTA is 66.4%, and 21 identity switches occur simultaneously, indicating that dense distribution and partial occlusion still have a certain impact on cross-frame identity association. In video 02, multiple cows are closely arranged along the feeding area, frequent overlaps occur between adjacent individuals with similar appearances, and cowshed rails and feeding facilities also cause partial structural occlusion. Therefore, the IDS of this video increases to 34, and the MOTP decreases to 68.9%, the lowest value among the five videos. This indicates that sufficient daytime illumination alone cannot guarantee good tracking performance, and occlusion caused by target density, individual spacing and cowshed facilities also affects the stability of target localization and identity association.

The cows in video 05 are also relatively densely distributed, accompanied by frequent spatial overlaps. Nevertheless, under relatively sufficient and stable daytime illumination conditions, this video achieves the highest IDF1 among the 5 scenarios, reaching 86.1%, and its MOTA, MOTP and HOTA are 82.3%, 80.3% and 71.9% respectively. This indicates that the model can maintain the identity consistency of most cows well. However, the IDS of this video still reaches 42, indicating that close contact and temporary mutual occlusion between cows can still trigger identity association errors in some frames. In contrast, video 04 presents more challenging nighttime monitoring conditions. Artificial light sources make some areas inside the cowshed relatively bright while other areas are relatively dark, forming relatively obvious uneven illumination. Under this scenario, the IDF1, MOTA and HOTA of video 04 are 77.1%, 73.7% and 61.5% respectively, all of which are the lowest values among the five videos, while its IDS increases to 53. Compared with video 03, the IDF1, MOTA, MOTP and HOTA of video 04 decrease by 5.0, 12.2, 7.6 and 11.9 percentage points respectively, while the IDS increases by 44. The results indicate that when uneven nighttime illumination interacts with factors such as target overlap and changes in cow activity states, it may reduce the continuity of object detection and increase the difficulty of cross-frame identity maintenance.

Although tracking accuracy varies across different scenarios, the processing speed of the model in the 5 videos remains at 43.1 to 44.8 FPS, all higher than the 30 FPS frame rate of the original surveillance videos. This result indicates that changes in illumination conditions, target density and occlusion degree mainly affect the accuracy of object detection and identity association, while having a relatively small impact on the computational efficiency of the model. Overall, stable illumination, clear target boundaries and larger individual spacing help improve tracking continuity and the reliability of identity association, while uneven illumination, dense distribution of cows, frequent occlusion and discontinuous target observation are more likely to cause trajectory interruption and identity switches.

#### 3.4.3. Comparison of Different Tracking Algorithms

**Figure 15:**
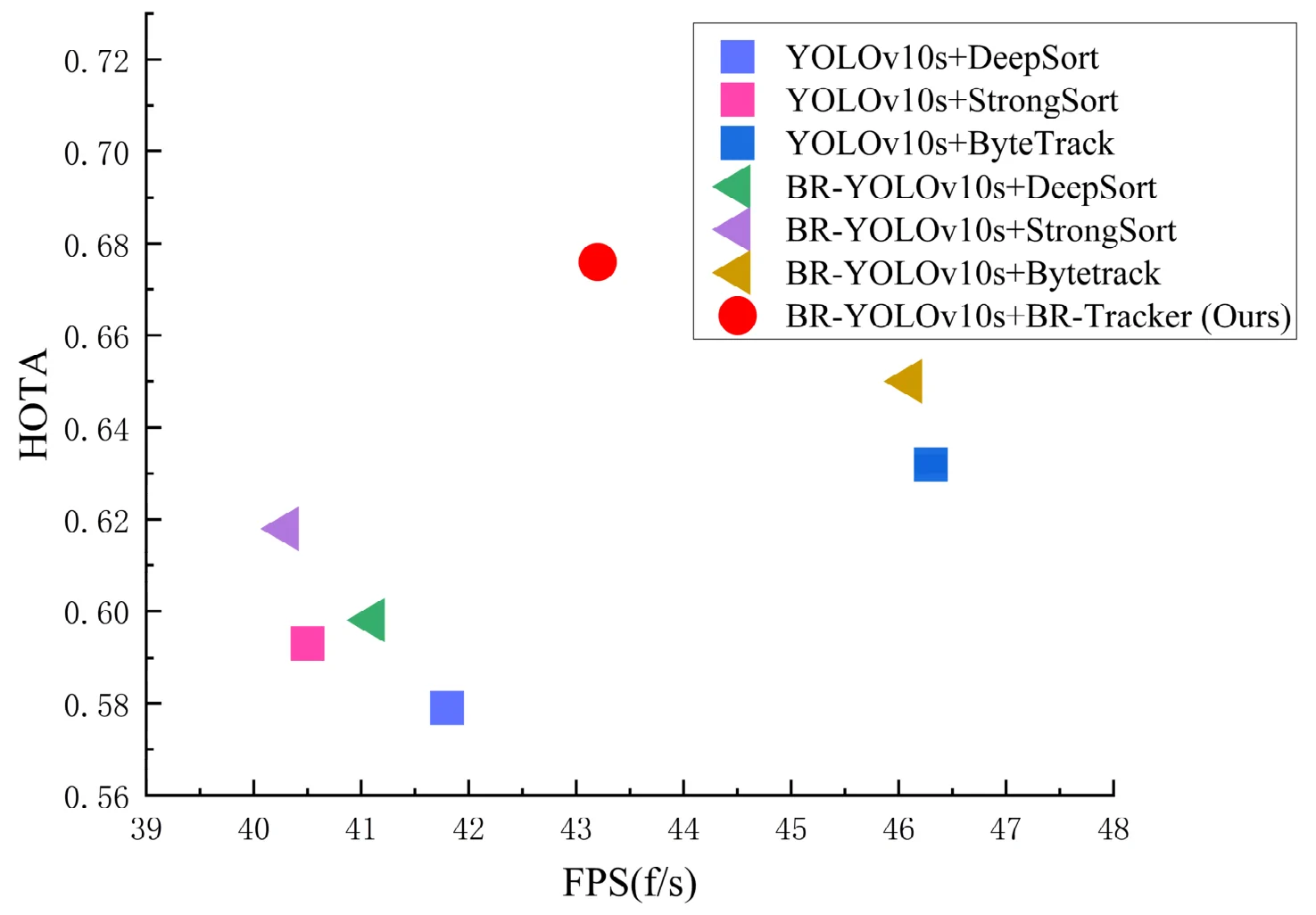
Comparison of the results obtained using different trackers.

To evaluate the performance of the cow multi-object tracking algorithm proposed in this paper, this study compares DeepSort [34], StrongSort [35], ByteTrack [33] and BR-Tracker on a dense cow MOT dataset. YOLOv10s and BR-YOLOv10s are combined with DeepSort, StrongSort and ByteTrack to analyze the impacts of detectors and tracking algorithms on the overall tracking performance. To ensure the fairness of comparative experiments, the re-identification (ReID) networks in DeepSort and StrongSort are specially trained using the cow dataset of this study, and the final MOT evaluation videos are not involved in the training of ReID networks. When detectors are replaced for comparison, the parameters of the same tracking algorithm and the ReID network weights of DeepSort and StrongSort remain unchanged. Each tracking configuration is repeatedly evaluated using detector weights trained with five different random seeds, and experimental results are reported in the form of mean ± standard deviation. As shown in Table 6, with the same detector, ByteTrack achieves higher IDF1, MOTA, MOTP and HOTA while producing fewer IDS, and its overall tracking performance is superior to that of ReID-based DeepSort and StrongSort. This is because different individual cows have high appearance similarity, and frequent occlusion leads to the loss of discriminative appearance information, thereby limiting the identity discrimination capability of ReID-based methods in intensive farming environments. After replacing YOLOv10s with BR-YOLOv10s, the main tracking accuracy metrics of all three tracking algorithms improve, while the IDS decreases, indicating that the improvement in detection performance can enhance subsequent trajectory association, but the degree of improvement varies with different tracking algorithms. When BR-YOLOv10s is used as the detector, BR-Tracker achieves the best overall tracking performance, with IDF1, MOTA and MOTP reaching 79.2 ± 0.4%, 80.5 ± 0.7% and 79.1 ± 0.5% respectively, while the IDS drops to the lowest value of 1446.4 ± 32.2. Compared with ByteTrack using the same detector, the IDF1, MOTA and MOTP of BR-Tracker increase by 2.5, 3.4 and 1.8 percentage points respectively, and the IDS decreases by 18.15%. The reason is that BR-Tracker adopts MPDIoU as the matching metric in the first-stage association. Compared with IoU, which only relies on the overlap degree of bounding boxes, MPDIoU further considers the geometric distances between corresponding corner points. When cow movement or occlusion causes the overlap degree of detection boxes to decrease, positions to shift or shapes to change, MPDIoU can more reliably evaluate the matching relationship between predicted trajectories and detection boxes, thereby reducing erroneous associations. As shown in Figure 15, compared with ByteTrack, which also uses the BR-YOLOv10s detector, the processing speed of BR-Tracker decreases from 46.1 FPS to 43.2 FPS, while the HOTA increases from 65.0% to 67.6%, reflecting that it achieves a good balance between overall tracking performance and processing efficiency. The above results further indicate that BR-Tracker is more suitable for intensive farming scenarios where cows are densely distributed, occlusion is frequent and individual appearances are highly similar.

**Figure 16:**
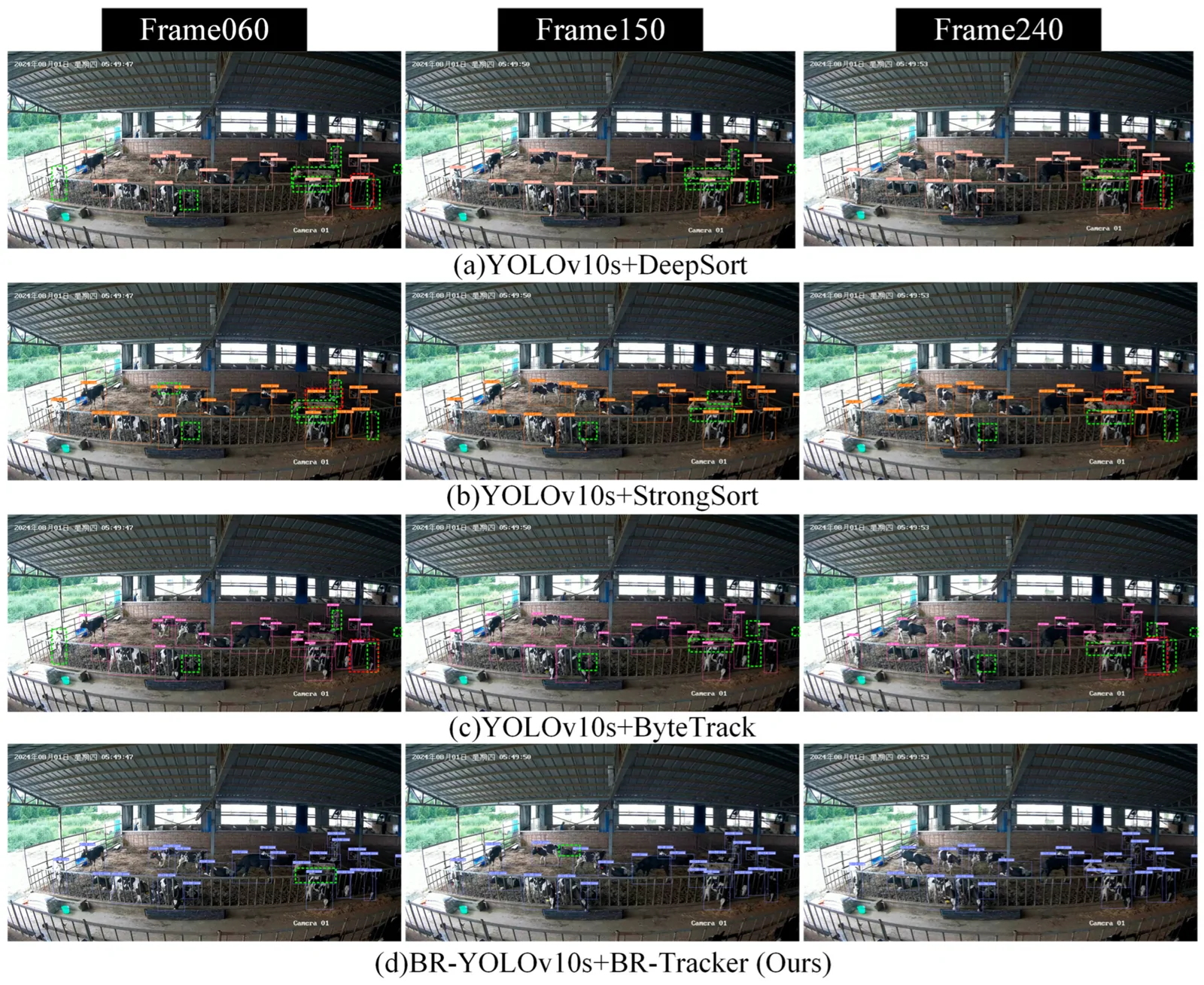
Qualitative comparison of tracking results obtained using different algorithms. Green dashed boxes indicate missed detections, while red dashed boxes indicate cows with identity switches. (The Chinese timestamp labels “年”, “月”, “日”, and “星期” denote the year, month, day, and day of the week, respectively).

The qualitative comparison results for the BR-Tracker algorithm proposed in this study with DeepSort, StrongSort and ByteTrack using the same surveillance video are shown in Figure 16. Frame060, Frame150 and Frame240 are all sampled from a continuous 6 s video segment at 3 s intervals and are used to analyze identity consistency and tracking continuity under conditions of dense cow distribution and partial occlusion. Green dashed boxes indicate missed detection targets, and red dashed boxes indicate cows with identity switches. As shown in Figure 16a, the DeepSort algorithm assigns a cow in the lower right area as ID-13 in Frame060. This target has no tracking output in Frame150, while in Frame240 this cow is reassigned as ID-27. In Figure 16b, the StrongSort algorithm also exhibits a similar problem. The cow marked as ID-24 in the right area in Frame060 loses its tracking box in Frame150 and is reassigned as ID-26 in Frame240. Although the DeepSort and YStrongSort algorithms can recover tracking output after the target reappears, they fail to maintain the original identity information, thereby reflecting that both algorithms still have certain limitations in identity maintenance and trajectory continuity after tracking interruption. As shown in Figure 16c, the ByteTrack algorithm also exhibits the identity switch problem after tracking interruption. The cow marked as ID-18 in the lower right area in Frame060 loses its tracking box in Frame150 and is reassigned as ID-35 in Frame240, failing to maintain the original identity when tracking is recovered. In addition, two other cows marked by green dashed boxes do not obtain tracking output in any of the three sampled frames, indicating that ByteTrack still has problems with target-missed tracking and unstable identity association in this scenario. In contrast, the BR-Tracker algorithm proposed in this paper in Figure 16d demonstrates better identity maintenance capability. The identity numbers of the tracked cows remain consistent across the three sampled frames, no identity switch phenomenon is observed, and the number of missed detection targets is significantly less than that of the other three algorithms. The above results indicate that in the demonstrated dense distribution and partial occlusion scenario, BR-Tracker can better maintain the consistency of cow identities and reduce tracking missed detections.

## 4. Discussion

This study proposes a BR-Tracker framework for multi-object tracking of dairy cows in intensive farming environments. In the object detection component, the RFADown module enhances the retention of local detail information during downsampling, which helps improve target feature representation under partial occlusion conditions. The PBRA module improves the network’s ability to distinguish densely distributed targets, while the SPPELAN structure enhances the fusion capability of multi-scale contextual features. The synergistic effect of these modules improves the object detection performance of the model in complex farming environments and provides more reliable target information for subsequent trajectory association. In the object tracking component, ReID-based tracking methods such as DeepSORT and StrongSORT mainly rely on appearance features for target association [34,35]. However, in intensive dairy farming environments, individual dairy cows have highly similar visual appearances and frequent partial occlusions, which reduces the discriminability of appearance features. To address this issue, this paper introduces MPDIoU into the matching stage. This metric considers not only the overlap degree between bounding boxes but also the geometric distances between corresponding corner points, which helps reduce mismatches caused by pose changes and bounding box shape variations. Experimental results show that the proposed method maintains generally stable tracking performance in the daytime and nighttime scenarios covered by this study, indicating that the collaborative optimization of the detection model and matching mechanism helps improve multi-object tracking of dairy cows in dense occlusion scenarios. The obtained continuous individual trajectories can also provide a basis for subsequent dairy cow behavior monitoring and precision farming management.

Nevertheless, this study still has certain limitations. First, the research data were collected from two dairy farms, both located in Henan Province, and the data from neither farm were used as a fully independent external test set. Considering that cross-regional data collection and frame-by-frame object and identity annotation require substantial labor, time, and coordination, this study has not yet been extended to more dairy farms. Therefore, the cross-farm generalization ability of the model still needs to be further verified. Second, the dairy cow breeds and feeding environments involved in this study are relatively homogeneous, and the applicability of the model under different dairy cow breeds, stocking densities, and cowshed layouts still needs to be further evaluated. On the current experimental platform, the processing speed of 43.2 FPS can meet the real-time detection and tracking requirements of a single 30 FPS video stream, but its real-time performance on low-computing-power edge devices or under parallel processing of multiple video streams still needs to be further verified. Finally, this study currently focuses only on object tracking in single-camera scenarios, and cross-camera identity association has not yet been investigated.

In response to the above limitations, future research will further expand the data sources and validation scope by introducing independent external data from different regions and dairy farms to build a more representative cross-scenario validation system, thereby enabling a more systematic evaluation of the generalization ability and stability of the model under different dairy cow breeds, stocking densities, and cowshed layouts. Meanwhile, while maintaining detection and tracking performance, the model structure and inference process will be further optimized to reduce computational overhead, thereby improving deployment efficiency on resource-constrained devices. In addition, research on cross-camera identity association will be gradually expanded to achieve continuous object identity association across different monitoring areas, thereby further enhancing the applicability of the model in practical large-scale farming environments.

## 5. Conclusions

This study proposes a deep learning-based multi-object tracking framework, BR-Tracker, to address the issues of missed detection and unstable tracking caused by densely distributed cows and frequent occlusions in intensive dairy farming environments. In the object detection stage, RFADown is introduced into the neck network of YOLOv10s to reduce the loss of locally visible features during downsampling. The SPPELAN structure is adopted to replace the original SPPF module to enhance the aggregation capability of multi-scale contextual features. Meanwhile, PBRA is introduced to improve the network’s feature representation and discrimination capability for densely distributed and partially occluded cow targets. In the object tracking stage, MPDIoU is introduced into the first-stage association process. It comprehensively considers the overlap degree of bounding boxes and the geometric distances between corresponding corner points, so as to improve the reliability of trajectory association when the positions and shapes of bounding boxes change due to cow movement and occlusion.

In object detection experiments, the precision, recall and mAP of BR-YOLOv10s reach 95.7 ± 0.3%, 90.3 ± 0.2% and 95.2 ± 0.2% respectively. Compared with the baseline YOLOv10s, the above metrics increase by 2.3, 1.9 and 1.3 percentage points respectively, and the parameter quantity and computational complexity of the model increase by 0.47 M and 2.3 GFLOPs respectively. The results of ablation experiments further indicate that RFADown, SPPELAN and PBRA play complementary roles in retaining local detail information, aggregating multi-scale contextual features and distinguishing densely distributed cow targets respectively. In the multi-object tracking evaluation based on 40 videos, the complete framework composed of BR-YOLOv10s and BR-Tracker achieves an IDF1 of 79.2 ± 0.4%, an IDS of 1446.4 ± 32.2, a MOTA of 80.5 ± 0.7%, a MOTP of 79.1 ± 0.5% and a HOTA of 67.6 ± 0.6%. Compared with the baseline combination of YOLOv10s and ByteTrack, IDF1, MOTA, MOTP and HOTA increase by 6.1, 5.7, 3.0 and 4.4 percentage points respectively, and IDS decreases by 23.19%. With the same BR-YOLOv10s detector, compared with ByteTrack, BR-Tracker increases IDF1, MOTA, MOTP and HOTA by 2.5, 3.4, 1.8 and 2.6 percentage points respectively and reduces IDS by 18.15%, indicating that the improved association mechanism helps improve trajectory association accuracy and identity maintenance capability. Compared with ByteTrack using the same detector, the processing speed decreases from 46.1 FPS to 43.2 FPS, but it is still higher than the 30 FPS capture frame rate of surveillance videos on the current experimental platform and can meet the real-time detection and tracking requirements of single-channel video.

Overall, the BR-Tracker framework proposed in this study exhibits favorable robustness and adaptability under complex breeding conditions such as densely distributed cows and partial occlusion. This method can provide technical support for intelligent cow monitoring and has potential application value in the field of precision livestock farming.

## Figures and Tables

**Table 1:** Comparison of experimental results of different detection algorithms.

Model	P (%)	R (%)	mAP (%)	Params (M)	GFLOPs
YOLOv5s	93.5 ± 0.5	87.2 ± 0.6	92.8 ± 0.4	7.13	16.5
YOLOv8s	95.1 ± 0.4	86.1 ± 0.5	93.7 ± 0.3	11.14	28.6
YOLOv10s	93.4 ± 0.4	88.4 ± 0.3	93.9 ± 0.3	7.23	21.6
YOLOv5s-CA [23]	93.8 ± 0.5	88.2 ± 0.5	93.2 ± 0.4	7.35	17.1
Pruned YOLO [27]	91.1 ± 0.6	85.7 ± 0.7	91.4 ± 0.5	11.9	25.1
BR-YOLOv10s (Ours)	95.7 ± 0.3	90.3 ± 0.2	95.2 ± 0.2	7.70	23.9

**Table 2:** Results of the ablation experiments and attention module comparisons.

Index	Models	P (%)	R (%)	mAP (%)	Params (M)	GFLOPs
1	YOLOv10s	93.4 ± 0.4	88.4 ± 0.3	93.9 ± 0.3	7.23	21.6
2	+RFADown	92.2 ± 0.6	88.7 ± 0.5	94.4 ± 0.4	7.36	22.1
3	+SPPELAN	93.2 ± 0.4	89.1 ± 0.3	94.1 ± 0.2	7.48	22.7
4	+PBRA	94.6 ± 0.5	88.9 ± 0.4	94.6 ± 0.2	7.32	22.3
5	+RFADown+SPPELAN	93.5 ± 0.5	89.8 ± 0.5	94.6 ± 0.4	7.61	23.2
6	+RFADown+PBRA	94.4 ± 0.4	89.6 ± 0.4	94.8 ± 0.2	7.46	22.9
7	+SPPELAN+PBRA	95.2 ± 0.3	89.4 ± 0.3	95.0 ± 0.2	7.56	23.3
8	+RFADown +SPPELAN+BRA	93.8 ± 0.6	88.9 ± 0.5	93.5 ± 0.6	7.65	23.5
9	+RFADown +SPPELAN+SK	92.5 ± 0.6	88.1 ± 0.7	92.0 ± 0.6	7.74	24.6
10	+RFADown +SPPELAN+CBAM	91.3 ± 0.7	87.5 ± 0.7	91.6 ± 0.8	6.65	22.8
11	+RFADown +SPPELAN+PBRA	95.7 ± 0.3	90.3 ± 0.2	95.2 ± 0.2	7.70	23.9

**Table 3:** Comparison of the impact of different detectors on tracking performance.

Model	IDF1 (%)	IDS	MOTA (%)	MOTP (%)	HOTA (%)	FPS
YOLOv5s+BR-Tracker	68.9 ± 1.2	1828.6 ± 75.6	76.2 ± 1.1	77.1 ± 0.7	58.9 ± 0.9	48.9
YOLOv8s+BR-Tracker	75.2 ± 0.7	1625.0 ± 61.8	77.1 ± 0.9	77.8 ± 0.7	63.4 ± 1.0	45.1
YOLOv10s+BR-Tracker	75.8 ± 0.7	1587.6 ± 61.2	78.5 ± 0.8	78.1 ± 0.7	63.8 ± 0.9	44.7
YOLOv5s-CA+BR-Tracker	70.6 ± 0.8	1745.2 ± 76.2	76.4 ± 1.0	77.5 ± 0.8	60.4 ± 1.0	47.6
Pruned YOLO+ BR-Tracker	73.4 ± 1.1	1691.8 ± 87.8	77.1 ± 1.1	77.8 ± 0.9	61.9 ± 1.2	44.2
BR-YOLOv10s+ BR-Tracker (Ours)	79.2 ± 0.4	1446.4 ± 32.2	80.5 ± 0.7	79.1 ± 0.5	67.6 ± 0.6	43.2

**Table 4:** Ablation results of the detector and tracker components.

Model	IDF1 (%)	IDS	MOTA (%)	MOTP (%)	HOTA (%)	FPS
YOLOv10s+ByteTrack	73.1 ± 1.1	1883.0 ± 76.2	74.8 ± 1.0	76.1 ± 0.8	63.2 ± 1.0	46.3
BR-YOLOv10s+ByteTrack	76.7 ± 0.8	1767.2 ± 50.8	77.1 ± 0.9	77.3 ± 0.7	65.0 ± 1.0	46.1
YOLOv10s+BR-Tracker	75.8 ± 0.7	1587.6 ± 61.2	78.5 ± 0.8	78.1 ± 0.7	63.8 ± 0.9	44.7
BR-YOLOv10s+ BR-Tracker (Ours)	79.2 ± 0.4	1446.4 ± 32.2	80.5 ± 0.7	79.1 ± 0.5	67.6 ± 0.6	43.2

**Table 5:** Tracking performance of BR-Tracker across five representative monitoring scenarios.

Video	IDF1 (%)	IDS	MOTA (%)	MOTP (%)	HOTA (%)	FPS
01	79.0	21	84.1	80.5	66.4	43.3
02	78.4	34	80.5	68.9	67.2	44.7
03	82.1	9	85.9	83.7	73.4	43.1
04	77.1	53	73.7	76.1	61.5	44.5
05	86.1	42	82.3	80.3	71.9	44.8

**Table 6:** Comparison of different tracking algorithms.

Model	IDF1 (%)	IDS	MOTA (%)	MOTP (%)	HOTA (%)	FPS
YOLOv10s+DeepSort	64.3 ± 1.4	2402.2 ± 91.5	72.6 ± 1.3	73.9 ± 1.1	57.9 ± 1.2	41.8
YOLOv10s+StrongSort	69.1 ± 1.3	2193.6 ± 82.1	73.2 ± 1.1	75.3 ± 1.0	59.3 ± 1.1	40.5
YOLOv10s+ByteTrack	73.1 ± 1.1	1883.0 ± 76.2	74.8 ± 1.0	76.1 ± 0.8	63.2 ± 1.0	46.3
BR-YOLOv10s+DeepSort	65.4 ± 1.1	2157.4 ± 75.3	73.3 ± 1.1	74.0 ± 0.9	59.8 ± 1.2	41.1
BR-YOLOv10s+StrongSort	69.2 ± 0.9	2045.8 ± 75.6	73.7 ± 0.9	75.4 ± 0.8	61.8 ± 0.8	40.3
BR-YOLOv10s+ByteTrack	76.7 ± 0.8	1767.2 ± 50.8	77.1 ± 0.9	77.3 ± 0.7	65.0 ± 1.0	46.1
BR-YOLOv10s+ BR-Tracker (Ours)	79.2 ± 0.4	1446.4 ± 32.2	80.5 ± 0.7	79.1 ± 0.5	67.6 ± 0.6	43.2

## Data Availability

The raw videos, annotated datasets, source code, model configurations, and trained weights are not publicly available due to project confidentiality requirements and data-use agreements with the participating dairy farms. Non-sensitive materials may be obtained from the corresponding author upon reasonable request, subject to approval.

## Appendix A

**Table A1:** Summary of the main training and tracking parameters used in this study.

Component	Parameter	Setting
Detector training	Input image size	640 × 640 pixels
Detector training	Batch size	16
Detector training	Number of epochs	200
Detector training	Optimizer	AdamW
Detector training	Momentum parameter	0.937
Detector training	Weight decay	0.0005
Detector training	Initial learning rate	0.01
Detector training	Learning-rate schedule	Linear decay
Detector training	Warm-up epochs	3
Detector training	Mosaic augmentation probability (mosaic)	1.0; disabled during the final 10 training epochs
Detector training	Horizontal flipping probability	0.5
Detector training	Random scaling parameter (scale)	0.5
Detector training	HSV augmentation (*hsv_h*, *hsv_s*, *hsv_v*)	0.015, 0.7, 0.4
Detector training	Model initialization	Corresponding pretrained weights
Detection filtering	Detection confidence threshold	0.30
Detection filtering	High-confidence detections	s ≥ 0.50
Detection filtering	Low-confidence detections	0.30 ≤ s < 0.50
First association	Association metric	MPDIoU
First association	Matching threshold	0.90
Second association	Association metric	IoU
Second association	Matching threshold	0.50
Track management	New-track initialization threshold	s ≥ 0.60
Track management	Next-frame matching threshold for track confirmation	0.70
Track management	Maximum lost-track buffer length	210 frames
Track management	Maximum retention duration	Approximately 7 s at 30 FPS
Kalman filter	State vector	(*x*, *y*, *a*, *h*, *v_x_*, *v_y_*, *v_a_*, *v_h_*)
Kalman filter	Time step	1
Kalman filter	Position standard-deviation weight	1/20
Kalman filter	Velocity standard-deviation weight	1/160
Repeated experiments	Number of independent runs	5 runs with different random seeds

## References

[1] Jiang B., Wu Q., Yin X., Wu D., Song H., He D. (2019). FLYOLOv3 deep learning for key parts of dairy cow body detection. Comput. Electron. Agric..

[2] Siachos N., Neary J.M., Smith R.F., Oikonomou G. (2024). Automated dairy cattle lameness detection utilizing the power of artificial intelligence; current status quo and future research opportunities. Vet. J..

[3] Van Hertem T., Tello A.S., Viazzi S., Steensels M., Bahr C., Romanini C.E.B., Berckmans D. (2018). Implementation of an automatic 3D vision monitor for dairy cow locomotion in a commercial farm. Biosyst. Eng..

[4] Lodkaew T., Pasupa K., Loo C.K. (2023). CowXNet: An automated cow estrus detection system. Expert Syst. Appl..

[5] Xudong Z., Xi K., Ningning F., Gang L. (2020). Automatic recognition of dairy cow mastitis from thermal images by a deep learning detector. Comput. Electron. Agric..

[6] Yu R., Wei X., Liu Y., Yang F., Shen W., Gu Z. (2024). Research on Automatic Recognition of Dairy Cow Daily Behaviors Based on Deep Learning. Animals.

[7] Alipio M., Villena M.L. (2023). Intelligent wearable devices and biosensors for monitoring cattle health conditions: A review and classification. Smart Health.

[8] Maroto-Molina F., Navarro-García J., Príncipe-Aguirre K., Gómez-Maqueda I., Guerrero-Ginel J.E., Garrido-Varo A., Pérez-Marín D.C. (2019). A Low-Cost IoT-Based System to Monitor the Location of a Whole Herd. Sensors.

[9] Wolfger B., Jones B.W., Orsel K., Bewley J.M. (2017). Evaluation of an ear-attached real-time location monitoring system. J. Dairy Sci..

[10] Song C., Jiang D., Wang F. (2023). Exploration of agricultural IoT breeding tracking based on a saliency visual target tracking algorithm. Turk. J. Agric. For..

[11] Lamanna M., Bovo M., Bellisola G., Romanzin A., Cavallini D. (2025). Rethinking wearable technology in dairy cows: Challenges and prospects for smart collars. Open Agric. J..

[12] Neethirajan S. (2020). Transforming the Adaptation Physiology of Farm Animals through Sensors. Animals.

[13] Mon S.L., Onizuka T., Tin P., Aikawa M., Kobayashi I., Zin T.T. (2024). AI-enhanced real-time cattle identification system through tracking across various environments. Sci. Rep..

[14] Myat Noe S., Zin T.T., Tin P., Kobayashi I. (2023). Comparing state-of-the-art deep learning algorithms for the automated detection and tracking of black cattle. Sensors.

[15] Liu H., Reibman A.R., Boerman J.P. (2020). Video analytic system for detecting cow structure. Comput. Electron. Agric..

[16] Tang H., Li Z., Zhang D., He S., Tang J. (2025). Divide-and-conquer: Confluent triple-flow network for RGB-T salient object detection. IEEE Trans. Pattern Anal. Mach. Intell..

[17] Li X., Wang W., Hu X., Yang J. (2019). Selective kernel networks. Proceedings of the IEEE/CVF Conference on Computer Vision and Pattern Recognition (CVPR), Long Beach, CA, USA, 16–20 June 2019.

[18] Woo S., Park J., Lee J.Y., Kweon I.S. (2018). CBAM: Convolutional block attention module. Proceedings of the European Conference on Computer Vision (ECCV), Munich, Germany, 8–14 September 2018.

[19] Zhu L., Wang X., Ke Z., Zhang W., Lau R.W. (2023). Biformer: Vision transformer with bi-level routing attention. arXiv.

[20] Han S., Fuentes A., Yoon S., Jeong Y., Kim H., Park D.S. (2023). Deep learning-based multi-cattle tracking in crowded livestock farming using video. Comput. Electron. Agric..

[21] Li G., Sun J., Guan M., Sun S., Shi G., Zhu C. (2024). A New Method for Non-Destructive Identification and Tracking of Multi-Object Behaviors in Beef Cattle Based on Deep Learning. Animals.

[22] Pretto A., Savio G., Gottardo F., Uccheddu F., Concheri G. (2024). A novel low-cost visual ear tag based identification system for precision beef cattle livestock farming. Inf. Process. Agric..

[23] Guo Y., Hong W., Wu J., Huang X., Qiao Y., Kong H. (2023). Vision-Based Cow Tracking and Feeding Monitoring for Autonomous Livestock Farming: The YOLOv5s-CA+ DeepSORT-Vision Transformer. IEEE Robot. Autom. Mag..

[24] Zhou H., Chung S., Kakar J.K., Kim S.C., Kim H. (2023). Pig Movement Estimation by Integrating Optical Flow with a Multi-Object Tracking Model. Sensors.

[25] Lu J., Chen Z., Li X., Fu Y., Xiong X., Liu X., Wang H. (2024). ORP-Byte: A multi-object tracking method of pigs that combines Oriented RepPoints and improved Byte. Comput. Electron. Agric..

[26] Mg W.H.E., Tin P., Aikawa M., Kobayashi I., Horii Y., Honkawa K., Zin T.T. (2024). Customized Tracking Algorithm for Robust Cattle Detection and Tracking in Occlusion Environments. Sensors.

[27] Zheng Z., Qin L. (2023). PrunedYOLO-Tracker: An efficient multi-cows basic behavior recognition and tracking technique. Comput. Electron. Agric..

[28] Wang A., Chen H., Liu L., Chen K., Lin Z., Han J., Ding G. (2024). Yolov10: Real-time end-to-end object detection. arXiv.

[29] Ma S., Xu Y. (2023). Mpdiou: A loss for efficient and accurate bounding box regression. arXiv.

[30] Zhang X., Liu C., Yang D., Song T., Ye Y., Li K., Song Y. (2023). RFAConv: Innovating spatial attention and standard convolutional operation. arXiv.

[31] Luiten J., Osep A., Dendorfer P., Torr P., Geiger A., Leal-Taixé L., Leibe B. (2021). Hota: A higher order metric for evaluating multi-object tracking. Int. J. Comput. Vis..

[32] Chattopadhay A., Sarkar A., Howlader P., Balasubramanian V.N. (2018). Grad-cam++: Generalized gradient-based visual explanations for deep convolutional networks. Proceedings of the 2018 IEEE Winter Conference on Applications of Computer Vision (WACV).

[33] Zhang Y., Sun P., Jiang Y., Yu D., Weng F., Yuan Z., Wang X. (2022). Bytetrack: Multi-object tracking by associating every detection box. arXiv.

[34] Pujara A., Bhamare M. (2022). Deepsort: Real time & multi-object detection and tracking with YOLO and TensorFlow. Proceedings of the 2022 International Conference on Augmented Intelligence and Sustainable Systems (ICAISS).

[35] Du Y., Zhao Z., Song Y., Zhao Y., Su F., Gong T., Meng H. (2023). Strongsort: Make deepsort great again. IEEE Trans. Multimed..

